# Three Ribosomal Operons of *Escherichia coli* Contain Genes Encoding Small RNAs That Interact With Hfq and CsrA *in vitro*

**DOI:** 10.3389/fmicb.2021.625585

**Published:** 2021-05-11

**Authors:** Thomas Søndergaard Stenum, Mette Kongstad, Erik Holmqvist, Birgitte Kallipolitis, Sine Lo Svenningsen, Michael Askvad Sørensen

**Affiliations:** ^1^Department of Biology, University of Copenhagen, Copenhagen, Denmark; ^2^Institute for Molecular Infection Biology, University of Würzburg, Würzburg, Germany; ^3^Department of Biochemistry and Molecular Biology, University of Southern Denmark, Odense, Denmark

**Keywords:** sRNA, CsrA, Hfq, ribosomal RNA operon, dual terminators

## Abstract

Three out of the seven ribosomal RNA operons in *Escherichia coli* end in dual terminator structures. Between the two terminators of each operon is a short sequence that we report here to be an sRNA gene, transcribed as part of the ribosomal RNA primary transcript by read-through of the first terminator. The sRNA genes (*rrA*, *rrB* and *rrF*) from the three operons (*rrnA*, *rrnB* and *rrnD*) are more than 98% identical, and pull-down experiments show that their transcripts interact with Hfq and CsrA. Deletion of *rrA, B, F*, as well as overexpression of *rrB*, only modestly affect known CsrA-regulated phenotypes like biofilm formation, *pgaA* translation and *glgC* translation, and the role of the sRNAs *in vivo* may not yet be fully understood. Since RrA, B, F are short-lived and transcribed along with the ribosomal RNA components, their concentration reflect growth-rate regulation at the ribosomal RNA promoters and they could function to fine-tune other growth-phase-dependent processes in the cell. The primary and secondary structure of these small RNAs are conserved among species belonging to different genera of Enterobacteriales.

## Introduction

Bacterial small regulatory RNAs (sRNA) are major post-transcriptional regulators of gene expression. Mechanistically, the majority of these sRNAs act by base paring to complementary sequences in mRNA targets, thereby altering translation initiation rates and/or mRNA stability (Wagner and Romby, 2015). Association rates between sRNAs and their target RNAs are often strongly increased by the presence of the homohexameric RNA chaperone Hfq, which binds both RNAs and facilitates base pairing (Santiago-Frangos and Woodson, 2018). Many sRNAs are involved in the rapid reorganization of bacterial gene expression as a response to various types of stresses (recently reviewed in Holmqvist and Wagner, 2017). However, sRNAs that are expressed in the absence of an acute stress have also been described, including anti-toxin sRNAs (reviewed in Brantl and Jahn, 2015), the sRNAs ChiX and Spot 42 which regulate different aspects of carbohydrate metabolism (Møller et al., 2002; Rasmussen et al., 2009; Beisel and Storz, 2011) and MgrR (Moon and Gottesman, 2009), a regulator of lipopolysaccharide composition. The sRNAs CsrB and CsrC are expressed in response to the accumulation of end-metabolism products at the entry to stationary phase (Lawhon et al., 2002; Gonzalez Chavez et al., 2010). In contrast to base-pairing sRNAs, CsrB/C act by sequestering a single protein target, the global regulator CsrA. This small (7 kDa) homodimeric RNA-binding protein acts by binding at or close to ribosome binding sites (RBS) in a myriad of different mRNAs (Potts et al., 2017). Targets of CsrA include mRNAs encoding proteins involved in carbon metabolism (Liu et al., 1995), biofilm formation (Jackson et al., 2002), motility (Wei et al., 2001), quorum sensing, and virulence (Altier et al., 2002). CsrB and CsrC function by mimicking CsrA targets and carry ∼18 and nine motifs for CsrA binding, respectively (Liu et al., 1997; Weilbacher et al., 2003). As a consequence, they antagonize CsrA by sequestering it away from its lower-affinity mRNAs targets, thereby decreasing the effective concentration of CsrA. More recent, similar activities on CsrA have been described for two additional sRNAs in *E. coli*; McaS (Jorgensen et al., 2013) and GadY (Parker et al., 2017), both of which are believed to contain two binding sites for CsrA. Unlike CsrB/C, both McaS and GadY also regulate gene expression independent of CsrA (Opdyke et al., 2004; Jørgensen et al., 2012; Thomason et al., 2012).

While the majority of characterized sRNAs from *E. coli* are transcribed from intergenic regions (IGRs) under the control of a dedicated promoter, several reports suggest that a substantial number of sRNAs are generated from 5′ or 3′ untranslated regions (UTRs) by RNase-dependent mRNA processing (Kawano et al., 2005; Chao et al., 2012; Miyakoshi et al., 2015). Additionally, the *glyW-cysT-leuZ* transcript, which is processed to give rise to tRNA^glyW^, tRNA^cysT^ and tRNA^leuZ^, also generates the sRNA 3′ETS^leuZ^ (Lalaouna et al., 2015). This sRNA, which is generated by RNase E-dependent processing, base-pairs to two other sRNAs, RyhB and RybB. The pairing neutralizes transcriptional noise from the *ryhB* and *rybB* genes and counteracts potential regulatory outcomes of inadvertent expression of the corresponding sRNAs. The 3′-ETS^leuZ^ is the first functional tRNA-derived fragment (tRF) described in bacteria. However, numerous tRFs have been reported in eukaryotes, where they control multiple different cellular processes, including genome stability (Martinez et al., 2017; Schorn et al., 2017), cell-cell signaling (Baglio et al., 2015), response to viral infection (Yeung et al., 2009) and stress responses (Emara et al., 2010; Saikia et al., 2014).

**FIGURE 1:**
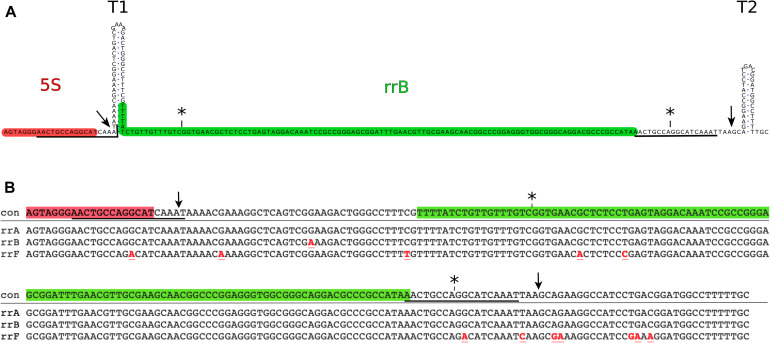
Genomic position of the *rrB* sequence and alignment of *rrA rrB* and *rrF*. **(A)** The *rrB* sequence found in *rrnB*. The 3′-end of the mature 5S transcript (highlighted in red) is followed by two transcriptional terminators (T1 and T2). An 18 base pair sequence (underlined) overlapping the 3′-end of the 5S gene, is found twice in the sequence with a spacing of 150 bp. The predominant form of RrB found on Hfq (RrB^short^) is highlighted in green (see also Figures 2, 3). The asterisks (*) above the sequence indicate the ends of the annotated version of *rrB* (Ecogene; Zhou and Rudd, 2012) and the arrows indicate the ends found by S1 mapping of the longer transcript (Figure 3). **(B)** Alignment of the sequences found in the 3′-end of three out of seven rRNA operons in *E coli.: rrnA (rrA), rrnB (rrB)* and *rrnD (rrF)*. con: consensus. Symbols are as in Figure 1A, red font indicates sequence differences compared to the consensus sequence.

In the present study, we have investigated the family of so-called tRNA-linked repeats (TLRs) from *E. coli.* The TLRs are a class of sequences located in tRNA or ribosomal RNA (rRNA) operons. Since the first description of TLRs in 1978 (Egan and Landy, 1978), a total of 22 TLR genes have been identified (Rudd, 1999), which are distributed between ten different loci on the *E. coli* K-12 chromosome, each locus harboring one to five TLRs. A striking feature common to all TLRs is that 18–19 bp of their 3′-end is identical to the 3′-end of the tRNA or rRNA gene that is located immediately upstream of the TLR (Figure 1). Regarding TLR functionality, one of the TLRs found in the pre-tRNA transcript *tyrT-tyrV* was initially reported to be involved in recovery from amino acid starvation (Bösl and Kersten, 1991). However, this claim was later retracted as the phenotype was shown to originate from a nearby open reading frame (Bosl and Kersten, 1994). Thus far, the TLRs have no known function. In the following, we present evidence that the three TLRs located downstream from rRNA operon A (*rrA*), B (*rrB*) and D (*rrF*), respectively, are transcribed, processed, and bind the post-transcriptional regulators Hfq and CsrA. We present evidence that these novel sRNAs may act as regulators to fine-tune CsrA activity.

## Materials and Methods

### Culture Growth and Media

The study was carried out in *E. coli* K-12 MAS1081 (MG1655 *rph^+^ gatC^+^ glpR^+^*). All strains used in the study are listed in Supplementary Table 1. Unless otherwise noted, all cultures were grown in MOPS minimal medium (Neidhardt et al., 1974) at 37°C shaking at 160 rpm and were grown exponentially for at least ten generations before start of the experiment to obtain balanced growth. Antibiotics were added as described for each experiment.

### RNA Purification and Northern Blotting

**FIGURE 2:**
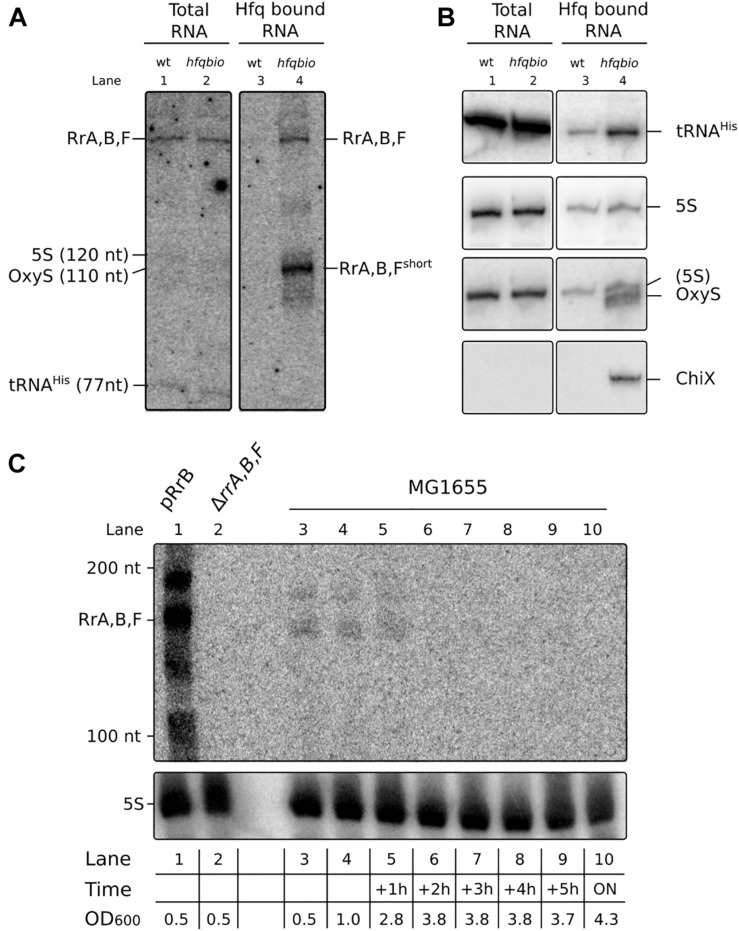
RrA, B, F is present as a distinct band in total RNA and a shorter version of RrA, B, F is enriched on Hfq. **(A)** Northern blot of a denaturing 10% polyacrylamide gel with RNA harvested from cultures of MG1655 (wt) or the isogenic Hfq-tagged derivative (*hfq_bio*). *E. coli* cultures were grown exponentially in MOPS minimal media supplemented with 15 mg/l chloramphenicol, 0.2% glucose, 10 mg/l uracil, 50 μM biotin. Both strains contain the plasmid pBirA. RNA was harvested directly from the cultures (lane 1 & 2) or from pull-down assays with Hfq (lane 3 & 4) as described in Materials and Methods. Equal volumes of the co-purified RNA were loaded in each lane to reflect equal numbers of input cells. **(B)** The same Northern blot membrane was probed and re-probed for tRNA^*his*^, 5S RNA, OxyS and ChiX to obtain the size markers indicated in panel **(A)** and as controls for the pull-down assay. The probe for OxyS was used after the 5S probe, and carryover signal from the 5S probe is clearly visible. Due to the low amount of RNA loaded in lane 1 and 2 (0.7 μg/lane) we did not detect OxyS or ChiX in the total RNA fraction. Lane 4 has 2.0 μg Hfq co-purified RNA, here we detect OxyS and ChiX but not in the control reaction with an untagged *hfq*, as expected. **(C)** Northern blot of a denaturing 6% polyacrylamide gel with RNA harvested from cultures of MG1655 + pRrB (lane 1), Δ*rrA, B, F* (lane 2) and MG1655 (lanes 3–10) as indicated. The cultures were grown exponentially in MOPS minimal media supplemented with 0.2% glucose. The first two samples of MG1655 were collected at OD_600_ values of 0.5 and 1, then samples were collected every hour for 5 h and the last sample was harvested after overnight (ON) incubation. The OD_600_ values for the culture at different harvest time points are shown in the table below the graph. The OD_600_ value for the pRrB and Δ*rrA, B, F* strains were both 0.5 at the time of harvest.

Culture aliquots were harvested into 1/4 vol ice-cold stop solution (95% ethanol, 5% phenol) (Bernstein et al., 2002). Subsequently RNA was purified using hot phenol and flash freezing in liquid nitrogen as in Fessler et al. (2020). Briefly: Stopped culture aliquots were centrifuged 2 min at 20.000 g and resuspended in 0.1 vol cold 0.3 M sucrose, 0.01 M NaOAc pH 4.5 followed by addition of 0.1 vol 2% SDS 0.01 M NaOAc pH 4.5. Phenol (saturated with water) was added to the liquid phase at a 1:1 ratio, the tubes were vortexed and incubated 3 min at 65°C. After freezing 15 sec in liquid N_2_ and centrifugation at 20.000 g for 5 min, the water phase was transferred to new tubes and the phenol extraction was repeated. If the RNA was used in an enzymatic reaction after purification, a chloroform extraction step was included. The RNA was ethanol-precipitated, washed by 96% ethanol, air dried at room temperature and dissolved in 10 mM NaOHAc, 1 mM EDTA. For northern blots, RNA was mixed 1:1 with loading buffer (0.1 M NaOAc (pH 5.0), 8 M urea, 0.05% (w/v) bromophenol blue and 0.05% (w/v) xylene cyanol) and size-separated on denaturing 0.4 mm thick polyacrylamide gels using 1 × TBE buffer (90 mM Tris, 90 mM boric acid and 2 mM EDTA). The RNA was electroblotted onto Hybond-N membranes (GE Healthcare) (1.5 V/cm, 1.5 h) in 40 mM Tris-acetate (pH 8.1), 2 mM EDTA. After UV-crosslinking (0.12 J/cm^2^) the membranes were pre-hybridized (1 h, rotating at 42°C) in hybridization solution (0.9 M NaCl, 0.05 M NaH_2_PO_4_ (pH7.7), 5 mM EDTA, 5 × Denhardt’s solution (0.1% BSA, 0.1% Ficoll 400, 0.1% polyvinylpyrrolidone), 0.5% (w/v) SDS and 100 mg/ml sheared, denatured salmon sperm DNA). Probe hybridization was done by adding 30 pmol of oligo-DNA, 5′-end labeled with ^32^P (overnight, rotating at 42°C). Subsequently, membranes were washed several times with 0.3 M NaCl, 30 mM sodium citrate, 0.1% SDS at 42°C. Radioactive signals were quantified on a PhosphorImager (Typhoon-GE Healthcare) using ImageQuant software as previously described (Sørensen, 2001; Stenum et al., 2017) and in case of very low signals (e.g., Figure 2C) the signal found in the estimated position of a band was used. Before re-probing, membranes were stripped by washing several times with 98°C, 15 mM NaCl, 1.5 mM sodium citrate, 0.1% SDS, until no more radioactive signal could be detected by a Geiger-Müller tube. Probe sequences used in this study are listed in Supplementary Table 4.

### Hfq Pull-Down Assay

RNAs that bind Hfq were isolated using an *E. coli* strain, where the *hfq* allele was tagged with a biotinylation sequence (Hfq_bio). We chose this tag as it is not positively charged, to reduce the risk that the tag might unspecifically bind negatively charged RNA. Hfq_bio was biotinylated by the biotin ligase BirA, and biotin’s high-affinity binding to avidin was used for purifying Hfq_bio (Kay et al., 2009). To ensure full biotinylation of Hfq_bio we introduced the plasmid pBirA where *birA* is under the control of an IPTG-inducible promoter. We found that production of sufficient BirA was achieved without induction of transcription by IPTG.

Wildtype and Hfq_bio cells both containing the pBirA plasmid were grown exponentially in MOPS medium supplemented with; 15 μg/ml chloramphenicol, 0.2% glucose, 10 μg/ml uracil, 50 μM biotin, at 37°C for at least 10 generations. At OD_436_ = 0.8 the cells were pelleted and washed in medium without biotin, re-suspended in 2 ml lysis buffer (50 mM Tris-HCl pH 7.5, 50 mM NaCl, 5% glycerol), lysed by sonication and centrifuged (20,000 *g*, 60 min). Total RNA was prepared from an aliquot of the cleared lysate, and the remaining lysate was transferred to fresh tubes containing 300 μl equilibrated SoftLinkTM Avidin Resin (Promega) and left overnight rotating at 4°C. Then, the resin was washed four times in lysis buffer as above and RNA was harvested by phenol extraction and ethanol precipitation. For total RNA and the Hfq-bound RNA, RNA corresponding to 0.05 and 10% of the total culture volume respectively (approximately 2,000 ng for Hfq-bound RNA from the Hfq_Bio strain and 700 ng for total RNA samples), was used for analysis by northern blotting.

### S1 Nuclease Mapping

Briefly, a [γ-^32^P]-ATP end-labeled DNA oligo antisense to the TLR area of interest was hybridized to total RNA and single-stranded overhangs were removed by addition of S1 nuclease. The resulting fragments were visualized on a denaturing polyacrylamide gel. If the synthetic oligo extends beyond the end of the target RNA, the position of the end can be determined by the number of nucleotides removed from the DNA oligo. S1 nuclease was used to map the RrB transcript of a strain harboring the plasmid pTSS1. RNA was harvested by hot phenol 1 h after IPTG induction. One pmol of 5′-end [^32^P] -labeled probe was hybridized to 30 μg of total RNA from the strain of interest. Hybridization was done in 50% formamide, 20 mM HEPES, 0.5 mM EDTA, 0.2 M NaCl, 0.05% (w/v) SDS and performed overnight in a thermocycler (68°C for 10 min, then the temperature was lowered to 54°C and decreased 1°C every 30 min until reaching 20°C). Digestion was performed by adding 300 μl 0.28 M NaCl, 50 mM NaOAc pH 4.6, 4.5 mM ZnSO_4_ along with 300 U/ml S1 nuclease (Thermo Fisher Scientific) and incubating at RT for 60 min. Samples were phenol/CHCl_3_ extracted, ethanol precipitated, size separated by electrophoresis on 7 M urea, 10% polyacrylamide sequencing gels and detected by autoradiography.

### Circular RACE Mapping

Circular RACE was used to map the isoform of RrB enriched on Hfq. Briefly, RNA purified from the Hfq_bio purification experiment was circularized using RNA ligase, reverse transcribed using random hexamer primers, PCR amplified twice using nested sets of *RrA, B, F*-specific primers, and subjected to deep sequencing. The site of circularization thus reveals both ends of the transcript. Mapping was carried out essentially as described by McGrath (2011) omitting the TAP treatment: 500 ng of RNA co-purified with Hfq was circularized in 1× buffer by adding T4 RNA ligase. Reverse transcription was carried out using Super Script III RT (Thermo Fischer) and primed by random hexamer oligos. The area of interest was amplified twice by PCR with two different sets of specific primers (cRACE rrB-2 1F + cRACE rrB-2 1R and cRACE rrB-2 2F + cRACE rrB-2 2R, see Supplementary Table 3). The PCR-library was sequenced on an Illumina Mi-seq by 300 bp paired-end sequencing. The resulting sequences were merged and subsequently listed by abundance. All reads that could not be merged were left out of the analysis.

### RNA Stability During Rifampicin Treatment

Cultures were grown exponentially at 37°C, shaking at 160 rpm for at least 10 generations in MOPS medium supplemented with 0.2% glucose and 10 μg/ml uracil. At an OD_436_ of 0.7, 3 × 15 ml culture aliquots were collected (time 0 min) and rifampicin was added to the remaining culture to a final concentration of 100 μg/ml. Aliquots of 15 ml were collected at 2.5, 5, 10, and 20 min post rifampicin treatment. Five percent spike-in culture overexpressing tRNA^*selC*^ was added to each sample aliquot as in Stenum et al. (2017). RNA was harvested using TRI-reagent (Sigma), as described by the manufacturer. The RNA was size-separated on polyacrylamide gels, northern blotted and probed as described above. RrB^short^ transcript levels were normalized to the tRNA^*selC*^ level for each sample.

### Structure Probing

The structure of RrB was investigated *in vitro* with and without Hfq.

Reactions (10 μl) containing 0.1 pmol of 5′-end [γ-^32^P] ATP-labeled transcript and 50 nM unlabeled *E. coli* tRNA were incubated with different concentrations of hexameric Hfq (Hfq_6_) (3, 1.5, and 0 μM) at 37°C for 100 min along with the relevant cleavage buffer. For Pb^2+^ cleavage: 1 × Structural Probing Buffer (Ambion AM2237), Pb^2+^ was added to a final concentration of 10 mM and samples incubated 1 min at 37°C. Control (C1) was without Hfq and Pb^2+^. RNaseIII cleavage: 1 × Short Cut MnCl_2_ buffer (New England Biolabs) and 0.002 units Short Cut RNaseIII (New England Biolabs), samples were incubated 20 min at 37°C. Control (C2) was without Hfq and RNaseIII. Control T1: 1 × Structural Probing Buffer (Ambion AM2237), sample was incubated at 95°C for 1 min, transferred to 37°C for 1 min. After addition of 0.05 U RNase T1 (Ambion AM2237) the sample was incubated at 37°C for 5 min. OH ladder: 1 × Alkaline Hydrolysis Buffer (Ambion AM2237), sample was incubated at 95°C for 5 min. All samples were cooled by addition of 200 μl ice-cold H_2_O and transferred to ice. The digested RNA was phenol extracted, ethanol precipitated, resuspended in 1 × loading buffer II (Ambion AM2237), and separated on an 8% polyacrylamide/urea/TBE gel. Radioactive signal from the dried gel was visualized on a PhosphorImager (Typhoon -GE Healthcare). Hfq protein used for all *in vitro* experiments was a kind gift from Anders Boysen.

### MS2 Affinity Purification, RNA-Seq and MS-MS

**FIGURE 3:**
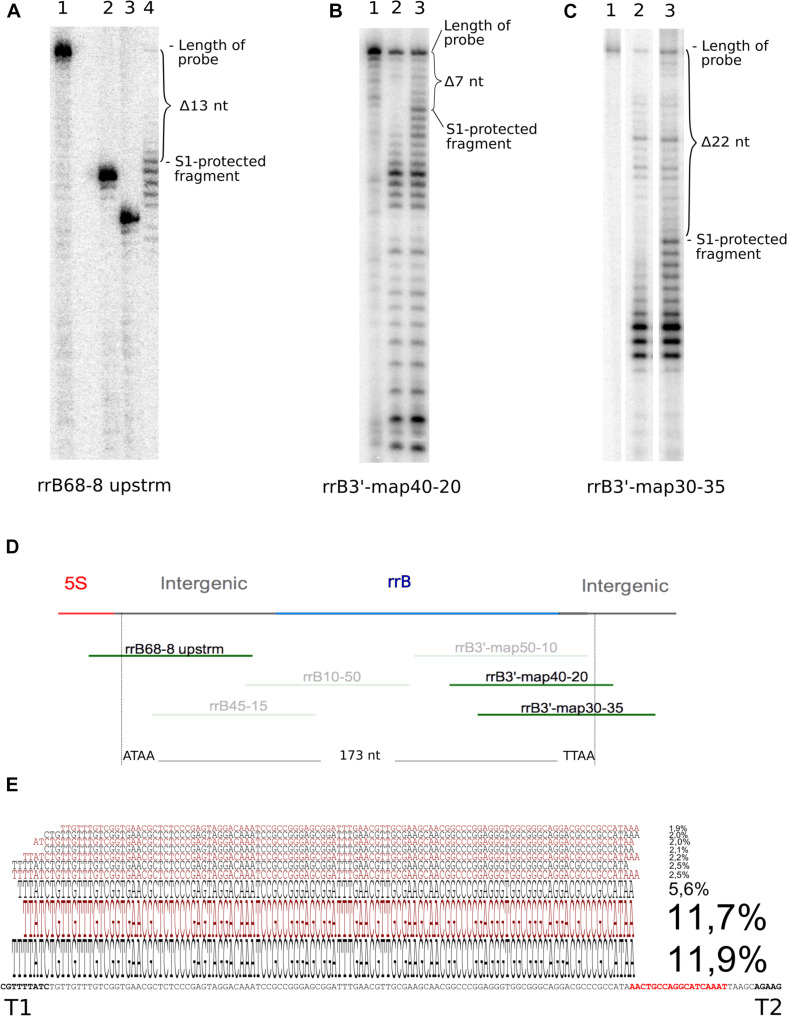
Determination of 5′ and 3′ ends of RrB by S1 nuclease analysis and of RrB^short^ by circular RACE. In the S1 nuclease mapping analysis **(A–C)** the ^32^P-labeled probe DNA was visualized by autoradiography of 10% poly-acrylamide sequencing gels. **(A)** mapping of the 5′-end using probe rrB68-8upstrm [shown in panel **(D)**] and total RNA from an IPTG-induced culture of MG1655 + pRrB over-expressing truncated ‘*rrfB and rrB*. The S1-protected fragment (lane 4) is 13 nt shorter than the untreated probe (lane 1). The exact number of nucleotides removed was determined by loading two labeled oligos as size markers [lane 2 (rrB54-8 upstrm) and 3 (rrB50-8 upstrm), probe rrB68- 8upstrm truncated by 14 and 18 nt respectively]. **(B)** probe rrB3′-map40-20 was hybridized to total RNA from an IPTG-induced culture of Δ22TLR + pRrfB-RrB over-expressing *rrfB* and *rrB*. The S1 protected band was shortened by 7 nt (lane 3) compared to the untreated probe (lane 1). Lane 2 shows a similar experiment using total RNA from the IPTG-induced strain Δ22TLR harboring empty vector (pJFR1). **(C)** Probe rrB3′-map30-35 was hybridized to total RNA from MG1655 + pRrfB-RrB over-expressing *rrfB* and *rrB*. The S1 protected band was shortened by 22 nt (lane 3) compared to the untreated probe (lane 1). Lane 2 shows a similar experiment using total RNA from the strain Δ22TLR harboring empty plasmid. **(D)** Map of the probes used for the experiments shown in panels **(A–C)**. Top line represents the genomic map; *rrfB* (5S) is shown in red, the intergenic sequences in gray and the annotated version of *rrB* in blue. Probe sequences are presented as green lines and probes shown in transparent colors were also used for mapping but detected no ends. The vertical broken lines denote the two ends detected in this study. **(E)** Circular RACE mapping of the RrB sequences that co-precipitated with Hfq. The ten most abundant RrB^short^ sequences detected by circular RACE and deep sequencing are aligned to the genomic sequence. The size of the characters correlates with their relative abundance, which is also stated as a percentage of total merged reads (*n* = 4722). Bold characters highlight the two terminators T1 and T2 and the direct repeat, which is also found at the 3′-end of the mature 5S RNA is highlighted in red (see Figure 1).

Affinity purification of MS2-tagged RNAs was done either with *in vivo* expressed RNA or *in vitro* transcripts that were added to the cell lysate. The 115 bp RrB^short^ sequence, mapped as the most abundant variant pulled down with Hfq (Figure 3) was cloned into the plasmid pNS21 using PCR amplifying RrB^short^ with either *Nhe*I or *Xba*I restriction sites on the ends. Purified PCR fragments were restriction digested with either *Nhe*I or *Xba*I and ligated into the pNS21 plasmid cut with the corresponding restriction enzyme, resulting in RrB^short^ 3′-fused to MS2 (*Nhe*I digestion) or RrB^short^ 5′-fused to MS2 (*Xba*I digestion), respectively. The transcripts from the resulting plasmids are terminated by the *vrrA* terminator originally found in *Vibrio* species. Transcription from the plasmids is under control of the P_*LlacO–*__1_ promoter. As the strains used for affinity purification harbor only the chromosomal copy of *lacI*, expression from these plasmids should be constitutive. This was verified by northern blotting (Supplementary Figure 4). Cell lysates of the strain *hfq*-FLAG (JVS814) harboring the different MS2-aptamer expressing plasmids, were prepared by growing cells in LB with 100 μg/ml ampicillin, to an OD_600_ of 1.0. Cell pellets corresponding to 50 OD_600_ units were resuspended in 800 μl of buffer A (20 mM Tris-HCl pH 8.0, 150 mM KCl, 1 mM MgCl_2_, 1 mM DTT) and lysed by addition of glass beads (0.1 mm), flash freezing in liquid N_2_ and shaking at 30 Hz for 10 min. Lysates were cleared by centrifugation (30 min 16,000 *g*, 4°C). Lysates corresponding to 2 and 0.5 OD_600_ units of cell culture were used to prepare RNA and protein, respectively. Before addition of lysate, the affinity chromatography columns (Bio-Spin #732-6008, Bio-Rad) were prepared by adding 100 μl amylose resin (New England Biolabs, #E8021S), washing three times with 2 ml buffer A and subsequently adding 200 pmol of MS2-MBP recombinant protein (a gift from the Jörg Vogel group). All steps of the affinity chromatography were done at 4°C. Following addition of the lysate, the columns were washed four times with 2 ml buffer A, and the RNA-protein complexes were eluted with 900 μl buffer A containing 12 mM maltose. RNA was purified using phenol-chloroform, followed by ethanol precipitation with the addition of 1 μl GlycoBlue (Thermo Fisher). Protein was harvested from the organic phase by acetone precipitation. RNA was subjected to next generation sequencing on the Illumina platform at the University of Würzburg. Proteins were detected and quantified by mass spec at the mass spec facility at University of Würzburg, see Supplementary Methods.

### Input Into Invenire CsrB/C Family RNA Prediction Algorithm

The web version of Invenire sRNA found at http://markov.math.umb.edu/inveniresrna/was used for the analysis. The dataset included the 85 sRNAs, 22 TLR sequences, and 2217 intergenic regions (<1,000 bp) annotated in the Ecogene database^1^ on November 24, 2017. In addition, 578 sequence peaks identified experimentally as CsrA binding sites by CLIP-seq (Potts et al., 2017) were included in the analysis.

### *In vitro* Transcription and Electrophoretic Mobility Shift Assay

Binding of RNAs to CsrA was examined using electrophoretic mobility shift assay (EMSA) with recombinant CsrA-3xFLAG (Supplementary Tables 1, 2) and *in vitro* transcribed RNA. DNA templates for *in vitro* transcription were made by PCR using pRrB, pRrB-3GGA and pCrsB (see Supplementary Tables 1, 2) as template for RrB, RrB-3GGA and CsrB, respectively. All primers used in the study are listed in Supplementary Table 3. Transcription was performed overnight at room temperature using the MEGAscript T7 transcription kit (ThermoFisher). The resulting RNA was treated with TurboDNase (ThermoFischer) (0.1 unit/μl, 30 min at 37°C), gel-purified from denaturing polyacrylamide gels, dephosphorylated using calf intestinal alkaline phosphatase (30 min at 37°C) and 5′ radiolabeled using T4 polynucleotide kinase and [γ-^32^P] ATP. After each step the RNA was phenol extracted once, chloroform extracted twice and ethanol precipitated. Binding reactions contained 100 mM KCl, 10 mM MgCl_2_, 2 mM DTT, 7.5% glycerol, 0.1 U SUPERase-IN RNase inhibitor (ThermoFisher), 2 ng total yeast RNA, 120 pM labeled RNA and 0–1,600 nM CsrA-3xFLAG in a 10 μl reaction. Samples were incubated 10 min at 37°C and separated on 8% native polyacrylamide gels using 1x TBE as buffer. Radioactive signals were detected on a PhosphorImager (Typhoon -GE Healthcare). The CsrA-3xFLAG was purified from the strain CsrA-3xFLAG carrying the plasmid pBAD-RBS-csrA:3xFLAG as described by Jorgensen et al. (2013).

### Construction of Deletion Mutants

For λ Red recombineering, we used a MAS1081-derivative strain (MAS1080) harboring the λ RED prophage [λ cI_857_ Δ(cro-bioA)] imported from strain HME68 (Sawitzke et al., 2007) by P1 transduction. Deletion mutations were constructed in this strain as described (Sawitzke et al., 2007). For each deletion, a *cat-sacB* cassette was first inserted at the desired genomic region by selecting for chloramphenicol resistance and confirming by PCR. The cassette was then replaced by a DNA fragment designed to yield deletion of 107 bp, 103 bp and 103 bp for *rrnA*, *rrnB* and *rrnD*, respectively, by counter-selection of the cassette by sucrose tolerance and confirmation by PCR and DNA sequencing. The deletions roughly match the annotated TLR sequences, including all GGA motifs found in RrA, B, F. For reasons outside the scope of this study, the DNA fragments were constructed so that the TLR sequence was replaced with a tRNA gene in two out of the three deletion sites (see Supplementary Table 1).

To obtain an *E. coli* mutant where the λ Red recombination enzymes had not been expressed, and thereby reduce the risk of undesired genome mutations, each mutated locus was then moved to an otherwise wildtype strain by P1 transduction first of each *cat-sacB* cassette and next of the locus carrying a deletion. P1 transduction was done as described by Miller (1972).

### Biofilm Measurements

Biofilm was measured in microtiter plates using peg-lids (Nunc-TSP, cat. no. 445497) and crystal violet staining. Cultures were grown in 96-well flat-bottom microtiter plates. Ten microliter outgrown culture was used to inoculate each well containing 150 μl YT media (per liter: 8 g tryptone, 5 g yeast extract and 5 g NaCl) supplemented with 100 μl/ml ampicillin and 1 mM IPTG for strains carrying plasmids. The outer-most wells on the plates were not used, as the results from these were found to fluctuate more than average. Plates were incubated at 37°C for 48 h (no shaking) to ensure all cultures were completely outgrown. After incubation the pegs were washed once in wash buffer (25 mM Tris pH 7.5, 100 mM NaCl) and placed in 0.01% crystal violet for 15 min. Then, the pegs were washed three times in wash buffer and transferred to a fresh microtiter plate containing 180 μl 96% ethanol in each well. When the crystal violet was completely dissolved, the A_590_ of each well was measured along with the OD_600_ of each well of the growth plate. The optical densities from the growth plate were used to normalize the absorbance signals from the stained biofilm.

### Motility Measurements

Mobility was measured on soft agar plates. One microliter outgrown culture was used to inoculate each plate by injection of 1 μl culture halfway into the agar in the center of the plate. Plates contained YT media (per liter: 8 g tryptone, 5 g yeast extract and 5 g NaCl) + 0.3% agar, and were supplemented with 100 μl/ml ampicillin and 1 mM IPTG for strains carrying plasmids. The plates were incubated approx. 16 h at 37°C before the spread of the bacteria was measured. Measurements were repeated several times with approximately 2 h in between to compensate for differences in the growth rates of different strains.

### Translational Reporters

The *glgC-gfp* in-frame fusion was constructed by replacing the *Nsi*I-*Nhe*I fragment of pXG10-SF (Supplementary Table 2) with the 5′UTR and first 30 nucleotides of the *glgC* coding sequence by restriction digestion and ligation. The *pgaA-lacZ* in-frame fusion was made by cloning the promoter of *lacI* along with a *Nhe*I restriction site into pGH253-kan (Supplementary Table 2) by replacing the *Eco*RI-*Bam*HI fragment. Subsequently, the 5′UTR and first 30 nucleotides of the *pgaA* coding sequence was inserted using *Bam*HI-*Nhe*I digestion. All primers used in the study are listed in Supplementary Table 3.

### Beta-Galactosidase Measurement

Beta-Galactosidase (β-gal) activity was measured from overnight cultures harboring the translational fusion *pgaA-lacZ*, grown in test tubes in YT media (per liter: 8 g tryptone, 5 g yeast extract and 5 g NaCl) supplemented with 15 μg/ml kanamycin. The β-gal activity was measured using the fluorescent substrate *4-methylumbelliferone b-D-galactopyranoside* (MUG), as described (Li et al., 2018). OD_600_ of the cultures was measured and used to normalize the corresponding β-gal values.

### GFP Measurements

Fluorescence of GFP was measured from strains harboring the *glgC*-*gfp* translational fusion. Cultures were grown in MOPS minimal media supplemented with 0.2% glucose, 15 μg/ml chloramphenicol and 100 μg/ml ampicillin when needed, at 37°C shaking at 300 rpm in 96-well black microtiter plates with clear, flat bottoms (Costar) in a plate reader (Synergy H1, Biotek). OD_600_ and GFP fluorescence (excitation 470 nm and emission 510 nm) was measured simultaneously every 10 min. The values for GFP were normalized to the corresponding values for OD_600_. The normalized data was plotted against time and the area under the curve was calculated as a measure of relative GFP expression. The same amount of data points were used for each strain and only data where cultures were growing exponentially was used in the analysis.

## Results

### Expression of RrA, B, F and Interaction With Hfq

Three out of seven ribosomal RNA (rRNA) operons from *E. coli* contain a sequence from the TLR-family downstream from the 5S gene (Supplementary Figure 1). These three sequences, named *rrA*, *rrB* and *rrF* respectively, are very similar in sequence (Figure 1). Furthermore, their location downstream of 5S, and their sequence, is highly conserved in many species of the Enterobacteriales, suggesting a specific role for these sequences and structures (Supplementary Figure 2).

We hypothesized that the sequences may function as sRNAs. To begin testing this hypothesis we first examined whether RNA molecules of defined sizes could be detected after processing of the primary *rrn* transcripts. Total RNA harvested from exponentially growing *E. coli* K-12 cultures was subjected to northern blot analysis. To detect RrA, RrB and RrF, here collectively referred to as RrA, B, F, we used a single probe, which is expected to detect all three sequences since they only differ at a few positions. As shown in Figure 2A (lane 1 and 2), a distinct band of 150–200 nt was observed, confirming that RrA, B, F can be detected after processing of the primary transcript. The size of the RrA, B, F RNA was estimated based on re-probing of the membrane for transcripts of known sizes (Figures 2A,B) and comparison to an RNA ladder (Figure 2C). Next, we asked whether the RrA, B, F RNAs interact with the RNA chaperone Hfq, like many well-characterized *E. coli* sRNAs (Bilusic et al., 2014). To enable pull-down of Hfq and analysis of co-purified RNAs, a biotinylation sequence (Beckett et al., 2008) was inserted in the chromosomal *hfq* gene. The C-terminally tagged Hfq protein (Hfq_bio) is functional, as shown by intact repression of an mRNA target by an Hfq-dependent sRNA in the tagged strain (Supplementary Figure 3). After affinity purification of Hfq_bio using a streptavidin resin, co-precipitated RNA was analyzed by northern blot analysis. Detection of the known Hfq-binding sRNAs OxyS and ChiX only in the co-precipitated RNA verified that Hfq-binding RNAs had been enriched in the Hfq pull-down assay (Figure 2B). Interestingly, the pull-down revealed that a shorter form of RrA, B, F (RrA, B, F^short^) was highly enriched on Hfq in addition to the band detected in total RNA (Figure 2A, lane 4). Further, to verify the identity of the bands on our northern blots found by the RrA, B, F, probe, we made a blot containing RNA from a strain overexpressing RrB from pRrB, a strain deleted for *rrA, B, F*, and several samples from the wildtype strain harvested at different stages of growth (Figure 2C). The blot verifies the identity of the bands and shows that RrA, B, F are only detected during exponential growth, where the rRNA operons are actively transcribed. During stationary phase, the transcriptional activity of rRNA operons is absent or very low (Baracchini and Bremer, 1991) but the 5S, 16S and 23S RNAs are stable molecules. Therefore the 5S rRNA detected during stationary phase in Figure 2C was transcribed during growth and serves as a qualitative loading control.

The ends of the longer RNA species were mapped using S1 nuclease protection assay (Berk and Sharp, 1977). As this assay was not sensitive enough to detect chromosomally expressed RrA, B, F, we used a strain overexpressing the 5S gene *rrfB* along with *rrB* from a plasmid. The S1 protected fragments are displayed in Figures 3A–C. Several bands spaced with one nucleotide intervals are seen, which is a common observation when using S1 nuclease transcript mapping (Green and Roeder, 1980; Aiba et al., 1981; Brosius et al., 1982) and is probably due to “end-nibbling” (Shenk et al., 1975). Thus, we define the transcript ends based on the longest protected bands. For the 5′ end the probe was shortened by 13 nt (Figure 3A). For the 3′ end the two different probes were shortened by 7 nt (Figure 3B) and 22 nt (Figure 3C), respectively. They both predict the same end. The 5′end of RrB was detected 3 nt downstream of the mature 3′end of the 5S transcript (see Figures 1, 3A). This site has been described as the initial RNase E processing site of the pre-5S RNA (Roy et al., 1983; Li and Deutscher, 1995) suggesting that the 5′ end of RrB is generated by RNase E cleavage. The 3′end of RrB was detected 13 nt downstream of its annotated 3′end (Figure 1). This 3′end was verified using two different probes (Figures 3B,C). These results predict that the most abundant RrA, B, F transcript in total RNA has a length of 173 nt, which is in good agreement with the northern blots shown in Figures 2A,C. We also carried out S1 analysis with probes antisense to the area between the two observed ends to detect any alternative transcript ends, but could not detect any (data not shown, the probes are shown in Figure 3D).

To determine the ends of the Hfq-enriched shorter version of RrA, B, F (RrA, B, F^short^) we used the circular RACE method (McGrath, 2011), coupled with next generation sequencing. As this method is PCR-based it requires substantially less input RNA than the S1 nuclease assay. Circular RACE yielded a variety of sequences mapping to *rrA, B, F*, of which the ten most abundant are shown in Figure 3E. The most abundant reads predict the Hfq-associated RrA, B, F^short^ transcript to be 113–115 nt in length. This is in accordance with our observations from northern blots (Figure 2).

**FIGURE 4:**
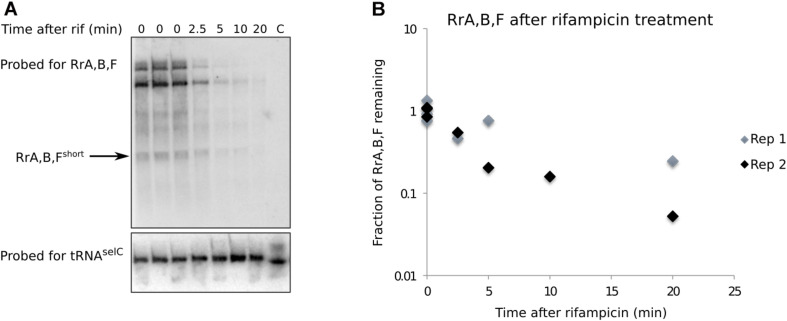
RrA, B, F^short^ is a shortlived transcript. **(A)** Northern blot made from total RNA harvested from a culture of MG1655. The culture was grown in MOPS minimal media supplemented with 0.2% glucose, 10 mg/l uracil. Rifampicin was added at 0 min to a concentration of 100 μg/ml. RNA was harvested at the indicated timepoints, right before and after rifampicin addition. Before RNA purification, spike-in cells overexpressing the tRNA gene *selC* were added to each experimental sample (see section “Materials and Methods”). 8–10 μg RNA was loaded in each lane. The blot was probed for RrA, B, F and tRNA^*selC*^ as indicated. The arrow indicates the band used for quantification. The lane marked “C” only contain spike-in cells. **(B)** Quantification of northern blots. The half-life of RrA, B, F was estimated as the average half-life calculated from two independent experiments (Rep 1, Rep 2). Rep 2 is shown in panel **(A)**. Independent biological replicates were obtained for all sample points except for the 10 min time point post rifampicin addition.

To detect the RrA, B, F^short^ fragment in the total RNA fraction, we carried out another northern blot analysis using substantially more total RNA per lane than the one presented in Figure 2A. This blot showed three different species of the RrA, B, F RNA (Figure 4A): the 115 nt RrA, B, F^short^, the 173 nt fragment, and a longer version. We suspect that the longer version represents a processing intermediate that includes the T2 terminator sequence. Thus, the RrA, B, F^short^ is not uniquely seen in the RNA fraction co-precipitating with Hfq but can also be detected in the total RNA fraction.

A short half-life of the RrA, B, F RNA could explain its low abundance relative to the rRNA transcripts expressed from the same operons and the absence of signal from stationary phase cells (Figure 2C). We measured the half-life of RrA, B, F^short^ by monitoring the levels of RrA, B, F^short^ upon transcription initiation blocking by addition of rifampicin to a culture in balanced growth (Figure 4). Indeed, we found that the half-life of RrA, B, F^short^ was ∼2.5 min, which is very short relative to the rRNA transcripts that are stable on the time scale of hours during exponential growth (Piir et al., 2011).

Taken together, we conclude that the RrA, B, F RNAs can be detected in exponentially growing cells as at least two transcripts of ∼173 and ∼115 nt that appear to interact with Hfq and that the shorter variant RrA, B, F^short^ has a half-life of about 2–3 min. The presence of RrA, B, F is therefore dependent on active rRNA transcription.

### The Structure of RrA, B, F Is Conserved Within the Order of Enterobacteriales

**FIGURE 5:**
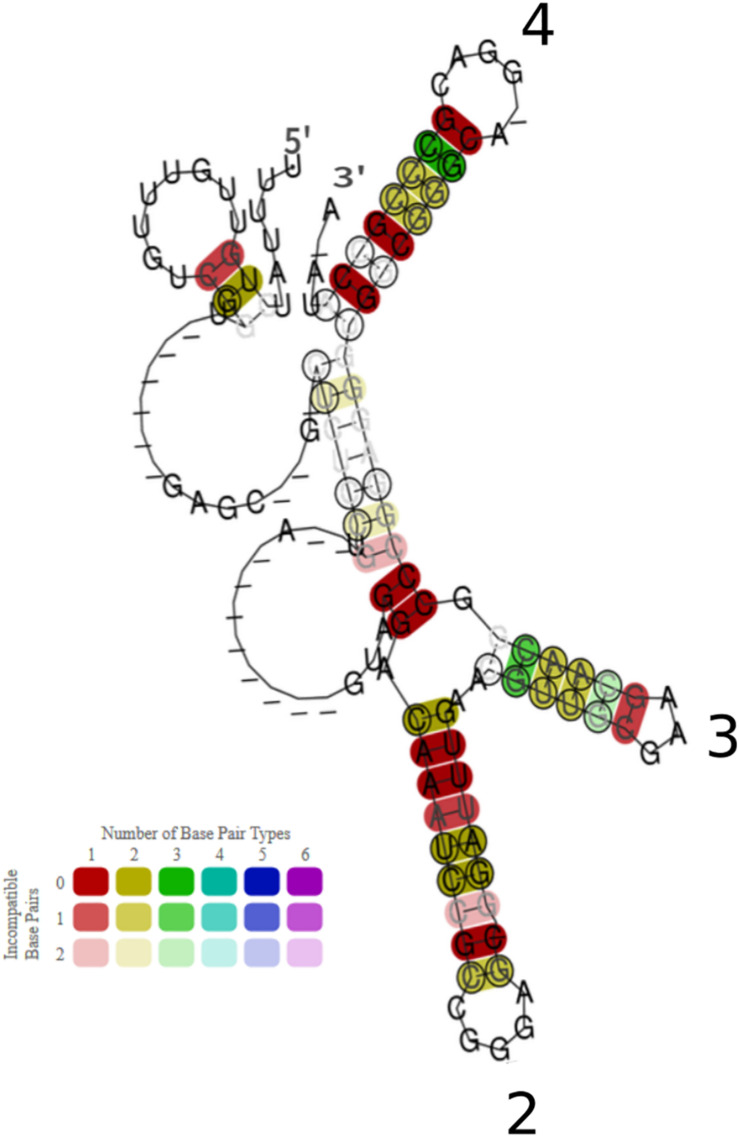
Conservation of sequence and structure of 30 RrB homologs found in Enterobacteria. The presented consensus structure was made using the algorithm LocARNA (Will et al., 2007, 2012; Raden et al., 2018) It is based on an alignment of 30 RrB^short^ homologs, from 30 different bacterial species, identified by BLAST search (see Supplementary Figure 2 and Supplementary Material to Figure 5). The base pairs of the locARNA structure are colored according to the level of structure and sequence conservation, as specified in the color legend. The hue indicates sequence conservation of the base pair (red: most conserved, purple: least conserved). The color saturation indicates the structural conservation of the base pair (High saturation: most conserved, low saturation: least conserved). Stem-loops are numbered as in Figure 6, Stem-loop 1 is not predicted to form in this consensus structure. Genera of the organisms included in the analysis are: *Salmonella, Shigella, Klebsiella, Escherichia, Pectobacterium, Brenneria*; the more distant: *Citrobacter, Enterobacter, Providencia, Raoultella*; and the most distant: *Cedecea, Serratia* and *Hafnia*.

In order to assess the conservation of RrA, B, F between species we did a BLAST search^2^ with *rrB* and the sequences surrounding the locus as input (see Supplementary Figure 2 and Supplementary Material). Both the sequence and the location of the *rrB* downstream of the gene encoding 5S was found to be conserved in at least 31 species from 12 genera, all belonging to the order of Enterobacteriales (Supplementary Figure 2). To evaluate the structural conservation of the RrA, B, F homologs we used the locARNA algorithm (Will et al., 2007, 2012; Raden et al., 2018) to predict a consensus structure (Figure 5). The consensus structure shows a high degree of structural conservation, particularly in the three stem loop structures named 2-4 in the figure, which could indicate a conserved function of the RNA.

**FIGURE 6:**
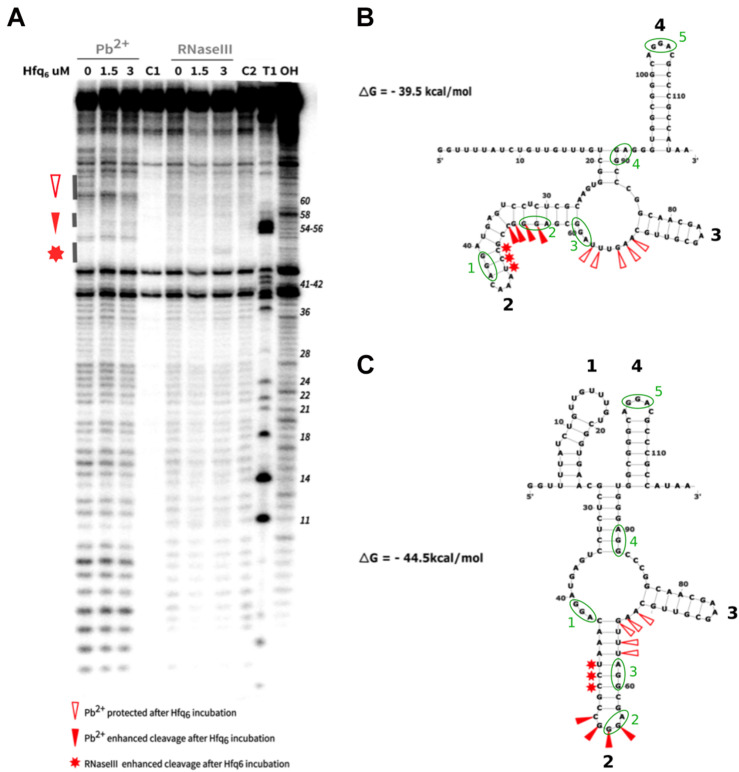
Structure probing of RrB^short^ with and without Hfq. The RrB^short^ *in vitro* transcript was incubated with 0, 1.5, or 3 μM of Hfq_6_ before addition of the cleavage agent. The partly digested RNA was size-separated on a denaturing 8% polyacrylamide gel. An autoradiogram of the gel is shown in panel **(A).** C1 indicates the transcript in Pb^2 +^ -buffer without addition of Pb^2 +^, C2 indicates the transcript in RNase III-buffer without addition of RNase III. T1 indicates the heated transcript treated with RNase T1 and OH indicates the transcript heated in alkaline buffer. Gray bars on the left side of the gel mark areas of Pb^2 +^ protection, Pb^2 +^ enhancement and RNase III enhancement. The nucleotide number with respect to the 5′ end is indicated at the right side of the gel. Intense bands at position 39 and 43 in all lanes can probably be attributed to background hydrolysis of the purified transcript before the analysis since they are abundant in all lanes. The structure of RrB^short^ was predicted by mfold with the constraint of a single-stranded region at nt 63-68 **(B)** or without any constraints **(C)**. The predicted free energies of the structures are indicated. Triangles and stars mark Hfq-dependent structural changes as indicated. GGA sequence motifs are marked by green ellipses; see section “RrB binds to CsrA *in vitro*.”

We speculated that the enrichment of RrA, B, F^short^ in RNA that had co-precipitated with Hfq could be due to a role for Hfq in chaperoning the correct folding of RrA, B, F, as shown for other RNAs (Geissmann and Touati, 2004; Soper and Woodson, 2008; Bordeau and Felden, 2014; Hoekzema et al., 2019). In this case, we would expect to see differences in the RrA, B, F^short^ structure with and without Hfq. To experimentally investigate the structure of the 115 nt RrB^short^ RNA, we performed RNA structure probing in the presence or absence of Hfq (Figure 6). As shown in Figure 1B, RrA^short^ and RrB^short^ are identical in sequence, and differ by only two nucleotides from RrF^short^. We therefore expect RrA, B, F^short^ to serve identical functions, and arbitrarily chose the 115 nt long RrB^short^ from the *rrnB* operon for these experiments. An *in vitro* transcript of the RNA was incubated with increasing concentrations of Hfq, followed by exposure to the RNA cleavage agent Pb^2+^ that primarily hydrolyzes single-stranded RNA (Ciesiołka et al., 1998), or the endoribonuclease RNase III that predominantly hydrolyzes double-stranded RNA with a strong preference for helices of sufficient length (Robertson, 1990). Fragmented RNA was size-separated on a polyacrylamide gel and visualized by autoradiography (Figure 6A) In general, only subtle changes in the fragment pattern were observed upon Hfq addition (Figure 6A). The most notable Hfq-induced change was found in the Pb^2+^-treated samples at the apparent single-stranded region at nt 63-64, 66-68. This sequence displayed reduced hydrolysis by Pb^2+^ in the presence of Hfq (Figure 6A). In contrast, nt 53-56 showed slightly enhanced Pb^2+^ cleavage, upon addition of Hfq. Finally, nt 48-50 displayed slightly enhanced RNase III cleavage after incubation with Hfq (Figure 6A). The change in cleavage pattern observed at nt 63-68 as a consequence of Hfq addition could be interpreted as direct binding of Hfq in the area, leading to protection from Pb^2+^-induced cleavage. Alternatively, the main outcome of interaction with Hfq may be a structural rearrangement that allows the region around nt 63-68 to engage in intramolecular base-pairing. The structure probing data do not allow us to distinguish between these two potential effects of Hfq. To pursue the hypothesis that Hfq may facilitate a structural rearrangement of the region, we used the algorithm Mfold (Zuker, 2003)^3^ to predict the most thermodynamically favorable RrB^short^ structure with (Figure 6B) and without (Figure 6C) the constraint of a single-stranded region from nt 63-68 (open red triangles). The predicted structure for RrB^short^ in the absence of the constraint (Figure 6C) forms stem loop #2 predicted by the consensus structure (Figure 5), whereas the predicted structure given the structural constraint of a single-stranded region from nt 63-68, does not (Figure 6B). The structure shown in Figure 6B is not the energetically most favorable one. This leads us to suggest that Hfq could be involved in folding of the RrA, B, F in order for the RNA to attain its most thermodynamically favorable structure, namely that shown in Figure 6C, which shows the consensus stem-loop structure (stem loop #2) predicted by locARNA.

### RrB Binds to CsrA *in vitro*

In order to identify potential interaction partners of RrA, B, F besides Hfq, we used MS2 affinity purification coupled with either mass spectrometry or RNA sequencing (Said et al., 2009; Corcoran et al., 2012; Lalaouna et al., 2015). Here, an RNA of interest is expressed as a fusion with an MS2-aptamer sequence, to allow purification of *in vivo* formed RNA-RNA or RNA-protein complexes using immobilized MS2 protein (Bardwell and Wickens, 1990; Said et al., 2009). We constructed plasmid-borne versions of RrB^short^, MS2-tagged at either the 5′-end or the 3′-end and affinity-purified the RNAs. Expression and purification of the tagged RNAs was verified by northern blotting (Supplementary Figure 4). RNAs co-purifying with RrB^short^ were identified by deep RNA sequencing (RNA-seq) and co-purifying proteins were identified by mass spectrometry. The results were compared to results for affinity purification of the MS2 RNA alone. While no specific enrichment was reproducibly detected in the RNA-seq analysis (data not shown), several proteins were specifically enriched in pull-downs with MS2-tagged RrB^short^, the most strongly enriched protein being the RNA-binding post-transcriptional regulator CsrA (Table 1). Somewhat surprising, Hfq was not found among the enriched proteins. We verified that RrB^short^ binds Hfq *in vitro* by electrophoretic mobility shift assay (EMSA). The EMSA analysis showed that RrB^short^ bound Hfq *in vitro* but with substantially lower affinity than the established Hfq-binding sRNA OxyS (Supplementary Figure 5). We will return to this point in section “Discussion.”

**TABLE 1:** Proteins identified by MS2-affinity purification to co-purify with RrB^short^.

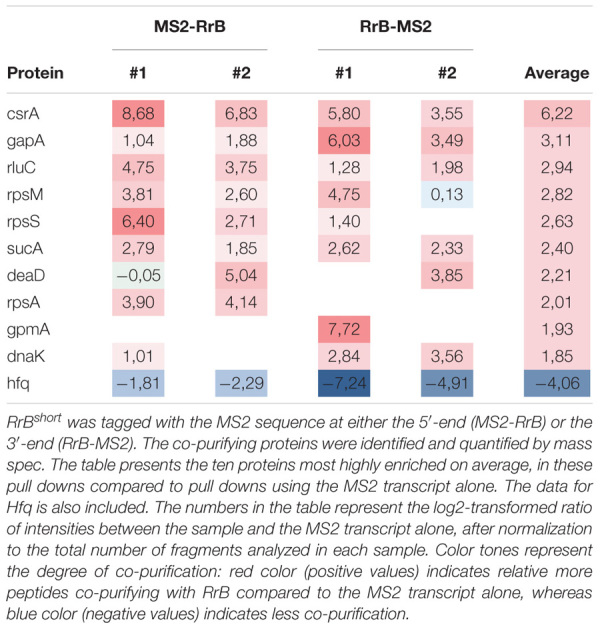

The canonical CsrA binding motif identified both in *S. typhimurium* (Holmqvist et al., 2016) and *E. coli* (Dubey et al., 2005; Potts et al., 2017) contains a GGA sequence located in the loop of a stem-loop structure. Interestingly, the RrA, B, F^short^ sequences have five GGA sequences, two of which are predicted to be located in the loops of stem-loop structures by the mfold and LocaRNA structure prediction algorithms (Figures 5, 6). Intriguingly, the structure probing experiments shown in Figure 6A suggest that the potential CsrA binding motif in loop 2 is only formed after interaction with Hfq.

The machine-learning-based algorithm InvenireSRNA (Fakhry et al., 2017) is designed to predict sRNAs of the CsrB/C family. To gauge how RrA, B, F rank in this algorithm compared to other *E. coli* non-coding RNAs, we provided the algorithm with a total of 2902 sequences, including all annotated *E. coli* sRNA sequences, the 22 TLR sequences, all sequences identified as CsrA-binding peaks from CLIP-seq data (Potts et al., 2017), and finally all intergenic regions of *E. coli* shorter than 1,000 bp. The results show that CsrB and CsrC both score high as expected (Table 2), while McaS and GadY both have low probability scores, suggesting that the features that make the latter acceptable binding partners for CsrA are not picked up by the algorithm. In accordance with the MS2-purification and structure prediction, RrA^short^, RrB^short^ and RrF^short^ are predicted to bind CsrA with high probability and, remarkably, rank among the 16 highest scoring sequences in the analysis (Table 2). Notably, the remaining 19 members of the TLR family located in tRNA operons all obtained a probability score of <0.01, suggesting RrA, B, F are unique among the TLR family in their affinity for CsrA.

**TABLE 2:** Transcripts predicted to regulate CsrA by the algorithm InvenireSRNA (Fakhry et al., 2017).

RNA	Probability score
CsrB sRNA	0.9997
csrB chip-SEQ	0.9977
speA chip-SEQ	0.9507
glyQ_ysaB	0.9505
setA_leuD	0,8794
OmpX chip-SEQ	0.872
lhr_grxD	0.8634
tatE_lipA	0,8518
CsrC sRNA	0.8435
zapB chip-SEQ	0.8308
csrC chip-SEQ	0.7979
ybhH_ybhI	0.7439
gltD chip-SEQ	0.7013
yqfE_argP	0.6407
RrA, B^short^	0.6245
RrF^short^	0.5833
McaS	0.005
GadY	0.0003

*The input into InvenireSRNA was all annotated sRNAs, the 22 TLR sequences, all sequences identified as peaks in a comprehensive CsrA CHIP-seq experiment (Potts et al., 2017) and all intergenic regions of *E. coli* shorter than 1,000 bp (2902 sequences in total). The table displays the 16 highest-scoring transcripts from the analysis, and the sRNAs McaS and GadY, along with the associated probability scores. Intergenic regions are named by the two genes flaking them, separated by “_” and CHIP-seq peaks by addition of “chip-SEQ” to the gene name. Note that CsrB/C are present twice as they are included as full-length sequences from the list of sRNAs and as peak regions from the CHIP-seq data.*

**FIGURE 7:**
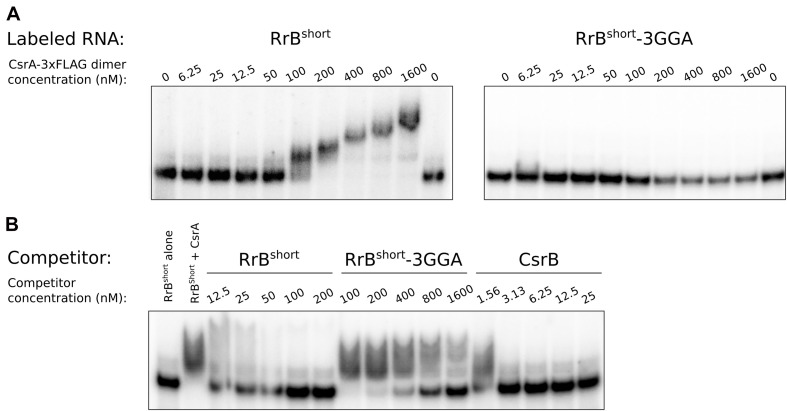
Electrophoretic mobility shift assays (EMSA) of RrB^short^ with CsrA. EMSA with 120 pM *in vitro* transcribed RNA and increasing concentration of CsrA-3xFLAG, as indicated. **(A)** EMSA of ^32^P-labeled RrB^short^ and an RrB^short^ mutant (RrB^short^-3GGA) where three out of five GGA motifs where replaced with ‘UUU’, mixed with increasing concentration of CsrA-3xFLAG, as indicated. **(B)** A competition assay with ^32^P-labeled RrB^short^ (120 pM), CsrA (200 nM) and increasing concentration of the *in vitro* transcribed unlabeled RNAs: RrB^short^, RrB^short^-3GGA and the well-known CsrA-binding RNA CsrB. Note the differences in concentrations between the different competitor RNAs. The first lane contains no CsrA in any of the experiments. The stated CsrA concentrations refer to the concentrations of the homodimer.

To further validate the binding between RrB^short^ and CsrA we conducted EMSAs. *In vitro* transcribed RrB^short^ was incubated with increasing amounts of purified CsrA-3xFLAG and separated on non-denaturing gels. As seen in Figure 7A, an RrB^short^-CsrA complex is observed from a concentration of 50 nM CsrA, and a complete shift is seen at 100–200 nM CsrA. At higher concentrations of CsrA, several complexes with higher molecular weights are visible. The number of different band sizes suggests that RrB^short^ has multiple binding sites for CsrA, which is expected based on our RNA structure predictions (Figures 5, 6). The binding relationship between CsrA and RrB^short^ is therefore complex but a first approximation of an overall K_D_ from our data in Figure 7A is around 100 nM. We repeated the binding experiment using a mutant version of RrB^short^, where three GGA sequences have been changed to “UUU” (number 1, 2 and 5 on Figure 6C), which includes the two motifs found in loops of stem structures. By only mutating the three GGA motifs found in single stranded regions, we do not expect this to have any consequences for the secondary structure of the RNA. The affinity for CsrA is clearly lower for this mutant compared to the wildtype RrB^short^ as the mutant does not show any shift within the tested range of CsrA concentrations. Note that part of the RNA is degraded at the highest CsrA concentration. We attribute this to residual activity of the RNase that was added during CsrA purification, see materials and methods. To further probe the specificity of RrB^short^ to CsrA we did competition binding experiments where a preformed CsrA-RrB^short^ complex (CsrA at 200 nM, RrB^short^ at 120 pM) was challenged with increasing concentrations of the unlabeled competitors RrB^short^, RrB^short^-3GGA and CsrB (Figure 7B). CsrB is believed to have ∼18 CsrA binding sites (Liu et al., 1997), a K_D_ around 1 nM of binding to CsrA (Weilbacher et al., 2003) and thus we expect it to be a more efficient competitor than RrB^short^. This is indeed what we observe, CsrB fully competes off the labeled RrB^short^ at a concentration of 3.13 nM, whereas a concentration of 50 nM is needed in the self-competition with RrB^short^. RrB^short^-3GGA show significantly less competition and is not able to fully compete the labeled RrB^short^ off CsrA within the range of concentrations tested.

With these binding assays we clearly show that RrB^short^ binds CsrA *in vitro* and that this interaction relies on the predicted binding sites located in single-stranded regions. The two remaining GGA motifs in the mutant RNA RrB^short^-3GGA do not efficiently bind CsrA on their own. We suspect that these low affinity GGA sites may require nearby high affinity sites in order to bind CsrA, this type of binding has previously been described for the CsrA homolog RsmE (Duss et al., 2014).

In combination, our results from MS2 affinity purification, structure prediction, the InvenireSRNA algorithm, and the EMSAs strongly suggest that RrB^short^, and likely RrA, B, F^short^, specifically interact with the post-transcriptional regulator CsrA.

### Phenotypic Effects of Altered *RrA, B, F* Levels

We next asked whether RrA, B, F might act by sequestering CsrA, similarly to the effects of CsrB, CsrC, GadY and McaS. To this end, we constructed an *E. coli* mutant deleted for the chromosomal copies of *rrA, B, F* (see Materials and Methods). Additionally, to overexpress *rrB* in a fashion that would presumably allow normal processing of RrB^short^, we cloned the *rrB* sequence including the 3′-half of the adjacent 5S gene and both terminators (T1 and T2) into a modified pUC18 plasmid (pJFR1, see Supplementary Table 2) to make pRrB.

**FIGURE 8:**
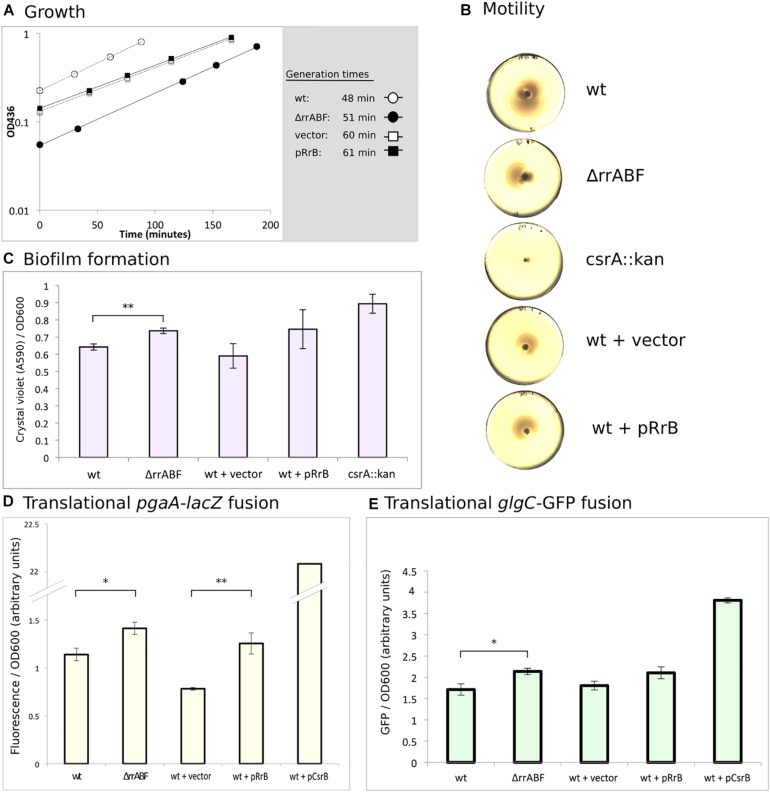
Phenotypes of deletion and overexpression of the *rrA, B, F* genes. **(A)** Growth of the four indicated strains. Cultures were grown in MOPS minimal media supplemented with 0.2% glucose and 100 μg/ml ampicillin for strains harboring plasmids. The indicated generation times were calculated as the average of two independent replicates. **(B)** Motility of the indicated strains was assayed on soft YT agar (0.3%) plates. Color and contrast of the photo has been manipulated to more clearly visualize the zone of bacterial growth. **(C)** Amount of biofilm formed on “peg-lids” by the indicated strains was quantified using crystal violet staining and absorbance measurements. **(D)** Quantification of the fluorescence produced by cleavage of 4-methylumbelliferyl-β-D-galactopyranoside (MUG) by β-galactosidase for outgrown cultures of the indicated strains. All strains carried a plasmid with a translational fusion of the *pgaA* leader to *lacZ* (pTSS36). **(E)** Quantification of GFP-signal for strains growing in exponential phase. All stains carried the translational fusion of the *glgC* leader to *sf-gfp* on a plasmid (pTSS16). In all cases, statistical probability was calculated using a two-sided student’s *t*-test and is indicated (^∗^*P* < 0.05, ^∗∗^*P* < 0.01).

Putative effects of RrB expression on the activity of CsrA were investigated by monitoring several CsrA-regulated phenotypes: growth rate, biofilm formation, motility, and post-transcriptional repression of the CsrA-regulated genes *pgaA* (Wang et al., 2005) and *glgC* (Baker et al., 2002). As a positive control, we moved a previously characterized *csrA::kan* allele (Romeo et al., 1993) into our wildtype strain (which is otherwise isogenic to the Δ*rrA, B, F* strain). The *csrA::kan* allele encodes a truncated version of CsrA which is known to have strongly reduced CsrA activity (Romeo et al., 1993). The results of these analyses are presented in Figure 8. In general, if RrA, B, F regulate CsrA activity in a manner similar to CsrB, we expect the phenotype of overexpression of *rrB* to display a similar tendency to that of the *csrA::kan* allele. Neither deletion nor overexpression of the *rrA, B, F* significantly affected the growth rate in minimal glucose medium (Figure 8A). For comparison, *E. coli* harboring a truncated CsrA have been reported to grow at approximately half the growth rate of the wildtype strain when glucose is the carbon source (Morin et al., 2016). The mutants lacking or overexpressing the *rrA, B, F* were also tested for motility on soft agar plates. Neither overexpression nor deletion of the *rrA, B, F* affected motility (Figure 8B). In contrast, the *csrA::kan* strain was severely impaired for motility as previously described (Romeo et al., 1993), although we note that this phenotype in our hands was very variable between replicate experiments.

CsrA is a negative regulator of PGA (poly-β-1,6-*N*-acetyl-d-glucosamine)-mediated biofilm formation (Wang et al., 2005). In agreement with this, we found that the *csrA::kan* strain produces more biofilm than wildtype as measured by crystal violet staining (Figure 8C). The Δ*rrA, B, F* strain also formed significantly more biofilm than the wildtype. However, overexpression of RrB also showed somewhat increased biofilm formation, although the difference from the strain carrying the empty vector control was not statistically significant.

In connection with biofilm, translation of the *pgaABCD* mRNA, encoding the machinery for synthesis and transport of PGA, is known to be strongly repressed by CsrA (Wang et al., 2005). Indeed, we found > 20-fold higher activity of β-galactosidase from a *pgaA-lacZ* fusion in the presence of CsrB overexpression than in the wildtype strain (Figure 8D). A modest increase in β-galactosidase activity was also observed in the Δ*rrA, B, F* mutant. Again, overexpression of RrB resulted in a phenotype similar to that of the Δ*rr,AB,F* mutant, pointing to a surprisingly similar effect on the *pgaA-lacZ* fusion of decreasing and increasing the RrA, B, F levels. There is good correlation between our data on biofilm formation and our data on the *pgaA-lacZ* fusion for all the strains (Figures 8C,D), as would be expected for CsrA-dependent regulation of biofilm (Wang et al., 2005).

Lastly, we tested the expression of GFP from a plasmid-borne translational *glgC*-*gfp* fusion during exponential growth, the growth phase where RrA, B, F accumulated (Figure 2C). The *glgCAP* operon encodes enzymes involved in glycogen metabolism, and is known to be repressed by CsrA (Baker et al., 2002). CsrB overexpression increases *glgC-gfp* expression substantially, while we observed slightly more GFP signal both upon deletion and overexpression of *rrA, B, F* (Figure 8E). In summary, we only observed minor phenotypic effects of changes in the expression of RrA, B, F. The RrA, B, F RNAs appear to modestly influence biofilm formation and expression from the CsrA-controlled *pgaA* and *glgC* mRNAs, but all three assays showed the same curious trend, namely that both deletion of *rrA, B, F* and overexpression of *rrB* resulted in a change consistent with modestly reduced CsrA activity. A plausible explanation for this unexpected similarity of phenotypes would be if RrB RNA expressed from the vector was processed into a form that exerted a different action on the tested properties than chromosomally expressed RrB. To address this hypothesis, we also tested expression of the *glgC-gfp* fusion in the presence of the pKK3535 plasmid that contains the entire *rrnB* operon, including *rrB*. Although the rRNA operon promoters are feedback-controlled and thus difficult to overexpress, this plasmid was expected to cause a relative increase in RrB levels, because the pKK3535 plasmid is responsible for approximately 50% of total rRNA synthesis even in the presence of the seven chromosomal rRNA operons (Steen et al., 1986). As shown in Supplementary Figure 5, overexpression of the *rrnB* operon resulted in increased *glgC-gfp* expression, and was thus consistent with reduced CsrA activity under this condition. In our hands, however, the strains containing pKK3535 had impaired growth rates (see Supplementary Figure 6A) and thus we did not pursue additional experiments with this plasmid.

### The Concentration of RrA, B, F Increases Upon Translational Halt

**FIGURE 9:**
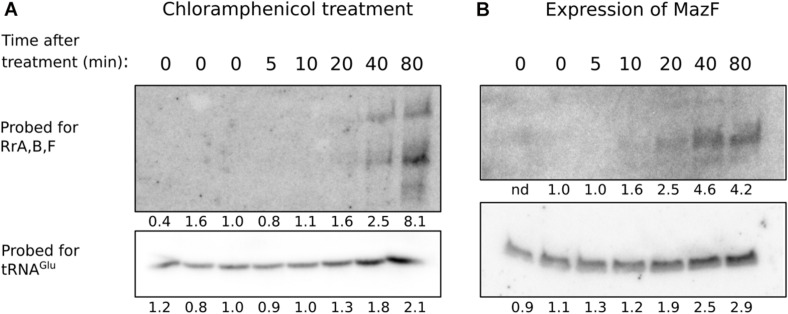
Levels of RrA, B, F after addition of chloramphenicol or induction of *mazF* expression. The strains MAS1081 **(A)** and TSS261 (MAS1081 + pMazF) **(B)** were grown in MOPS minimal media supplemented with 0.2% glucose or 0.4% glycerol, respectively. Treatment was induced by addition of either chloramphenicol (20 μg/ml) **(A)** or arabinose (0.1%) **(B)**. RNA was harvested at the indicated times after treatment. Equal volumes of RNA (6–15 μg/lane) were separated on 6% denaturing polyacrylamide gels and northern blotted. The blots were probed for tRNAGlu and RrA, B, F as indicated on the figure. Quantification of the radioactivity signal in each lane is given relative to the average value of the steady-state levels and is shown below each lane. nd: not determined due to high background.

The very modest effects of RrA, B, F on CsrA-controlled phenotypes suggest that if there is a physiological consequence of RrA, B, F interaction with CsrA then we might not have examined it under the proper growth conditions. In *E. coli*, rRNA expression is controlled by the second messenger ppGpp (Zhang and Bremer, 1995). A decrease in ppGpp levels leads to increased rRNA expression (as observed upon treatment with antibiotics specifically blocking translation (Kurland and Maaløe, 1962; Muto et al., 1975)). To test whether the ppGpp effect also applies to *rrA, B, F*, we induced translational arrest, either by chloramphenicol addition, or by expression of the toxin MazF, and investigated the level of RrA, B, F by northern blot analysis at several time points after induction. Chloramphenicol blocks translation by binding to ribosomes and inhibiting the peptidyl transferase activity (Das et al., 1966) while MazF leads to translational arrest by disrupting ribosome biogenesis and cleaving mRNAs (Culviner and Laub, 2018), thereby removing the template for translation. In both cases of translational arrest, we detect a strong increase in RrA, B, F transcript levels 20–80 min after the treatment (Figure 9). As an independent indicator of transcription from the rRNA operons, we also investigated the level of tRNA^*Glu*^. This tRNA is exclusively expressed from genes located in four rRNA operons (*rrnB, rrnC, rrnE, rrnG*), so we expected its expression pattern to resemble that of RrA, B, F. Indeed, the level of tRNA^*Glu*^ also increases during translational arrest, albeit not nearly to the same extent as RrA, B, F (Figure 9).

## Discussion

We identify three small genes that are co-transcribed from rRNA operons and processed to transcripts of defined lengths that bind the post-transcriptional regulator CsrA both *in vitro* and *in vivo*. As the transcription rate of rRNA is strictly correlated with the growth rate of *E. coli* (Potrykus et al., 2011), it is tempting to speculate that such short-lived sRNAs transcribed along with rRNA could function to align growth-rate-regulated transcription rates with other growth-phase-dependent processes in the cell, such as those regulated by CsrA. The relatively short half-life of RrA, B, F compared to other sRNAs makes biological sense if their abundance should reflect the transcriptional activity of the rRNA operons in real time.

We find a modest effect of overexpression of *rrB* on three of the CsrA-regulated phenotypes tested (Figure 8). The phenotypes observed upon overexpression of *rrB* agree with those expected from a mutant with modestly reduced CsrA activity. Such an apparent inhibition of CsrA upon overexpression of *rrB* could be due to binding of RrB to CsrA, resulting in regulation by titration in the classical way first described for CsrB/C. Direct binding between CsrA and RrB^short^ is experimentally supported by the results of our RNA affinity purification and EMSA analyses as well as theoretically supported by the InvenireSRNA prediction and the presence of consensus CsrA-binding motifs in the RrB^short^ structure. The fact that deletion of the *rrA, B, F* genes showed similar phenotypes to RrB overexpression is then counterintuitive (see Figure 8). Potentially, the chromosomally encoded form of RrB could fulfill a function for which the version of RrB expressed from our multicopy plasmid has a dominant negative effect (overriding the phenotype of the wildtype allele), in which case similar phenotypes of the chromosomal *rrA, B, F* deletions and the RrB overexpression could be expected. The curious effect or RrB overexpression was investigated further by introducing a plasmid expressing the entire *rrnB* operon (Supplementary Figure 6), which confirmed the results obtained by specific overexpression of RrB (Figure 8E). Since overexpression of *rrB* by two different cloning tactics affected the *glgC-gfp* fusion similarly, we conclude that deletion and overexpression of RrB both result in phenotypes consistent with reduced CsrA activity, but cannot currently provide a mechanistic explanation for this curious observation.

While we have demonstrated an interaction between the RrA, B, F RNAs and CsrA, deletion and overexpression of *rrA, B, F* show only modest changes of the CsrA-related phenotypes tested in this study. We want to note that CsrA is the major hub in a regulatory network with many inputs (Romeo and Babitzke, 2018). For that reason, one might not expect that the absence, or presence in excess, of RrA, B, F would lead to prominent phenotypes, but merely fine tuning of the activity of CsrA. Further, EMSA analyses (Figure 7) showed that CsrB totally competed RrB^short^ off CsrA at a ten-fold lower concentration than RrB^short^ itself (Figure 7B). This shows that CsrB is a higher-affinity CsrA binding partner than RrA, B, F and that we therefore might expect small effects, if any, of changes to the RrA, B, F levels in situations where CsrA-regulation by CsrB/C is at play.

Our northern analysis shows that RrA, B, F are most abundant when the rRNA operons are expressed, namely during exponential growth (Figure 2C), and especially upon treatments that lower ppGpp production by arresting translation (Figure 9). During translational halt, RrA, B, F levels increased more than the tRNA^*glu*^, which is also expressed solely from rRNA operons. This difference in the extent of induction could either mean that there is differential expression of the genes in the rRNA operons upon translational arrest, or, more likely, translational arrest may result in increased stability of the RrA, B, F sRNAs relative to tRNA^*glu*^ by an unknown mechanism. Our data do not allow us to distinguish between these possibilities. Nevertheless, the experiments show that conditions exist in which the abundance of the RrA, B, F RNAs increase substantially. If they play a greater role in cell physiology than the fine-tuning of CsrA-regulated phenotypes shown in Figure 8, their impact should maybe be sought under such conditions, or at least under conditions where CsrB/C concentrations are low but the transcription rate of rRNA is high.

We find that *rrA, B, F* is well conserved in various different genera of bacteria including numerous pathogenic species. It is well known that growth-phase-dependent regulation is important for bacterial virulence (Dalebroux and Swanson, 2012; Kitamoto et al., 2016). Thus, if RrA, B, F is used as an indicator of growth rate in the cell, it could also potentially have a regulatory effect on the pathogenesis of these virulent stains.

A hallmark of all the members of the TLR family found in *E. coli* is the presence of a short (18–19 bp) repeated sequence identical to the 3′-end of the mature tRNA or rRNA from the same operon. For the tRNA or rRNA this 3′-end sequence is important for processing (reviewed in Mackie, 2013). The 3′-end of the 173-nt form of RrB was mapped 3 nt downstream of the repeated sequence that is identical to the final 18 bp of mature 5S RNA. As this repeated sequence and structure is known to be important for the RNase E cleavage immediately downstream of 5S, we suggest that the repeat in the RrB 3′-end is also recognized by RNase E, and that this explains the function of the repeated sequence.

RrB^short^ was enriched on Hfq in the co-purification experiment (Figure 2), and showed specific binding to Hfq by EMSA analysis (Supplementary Figure 5), which could suggest a role for Hfq in folding or processing of RrB. Hfq could affect folding and the kinetics of the processing but is not essential for processing since RrB^short^ was also observed in a Δ*hfq* mutant (Supplementary Figure 7). Alternatively, the enrichment could reflect that RrB^short^ functions as a base-pairing sRNA to regulate one or more target RNAs, chaperoned by Hfq. GadY (Parker et al., 2017) and McaS (Jorgensen et al., 2013) are two recent examples of dual function sRNAs that can both function to bind and titrate out CsrA, and function as generic Hfq-dependent base-pairing sRNAs to regulate target mRNAs. Although we cannot rule out either option, we favor the former option, namely that Hfq participates in the folding or processing of RrB, which is also supported by our structure probing with and without Hfq (Figure 6). If Hfq assisted with pairing of RrB^short^ to a target RNA(s), we would have expected to detect the binding between RrB^short^ and Hfq in the mass spectrometry analysis of the proteins co-purifying with RrB^short^, and the RNA-seq analysis could have revealed the target RNA(s). On the other hand, if the role of Hfq is to facilitate the refolding or processing of RrB, then the affinity of Hfq for the refolded product RrB^short^ need not be very high, why we speculate that competing binders with higher affinity, like the abundant CsrA protein, may have excluded binding of Hfq to RrB^short^ in the MS2-RNA affinity purification experiments.

The *rrA, B, F* genes are found downstream of the gene encoding 5S in three out of seven rRNA operons of *E. coli.* These three operons are furthermore the only three that contain two terminator sequences (T_1_ and T_2_), whereas the remaining four rRNA operons have a single terminator (Supplementary Figure 1; Lesnik et al., 2001). The significance of the dual terminators is not clear and has been puzzling since its discovery (Brosius et al., 1981; Brosius, 1984; Ghosh et al., 1991; Orosz et al., 1991). The termination efficiencies of each of the terminators from the *rrnB* operon have been measured. T1 was measured to terminate 87% of transcripts while T2 terminated 100% of transcripts (Orosz et al., 1991). As the *rrA, B, F* genes are located between the two terminators we suggest that this dual terminator arrangement could serve the purpose of regulating the expression of *rrA, B, F*. In this way, the expression of *rrA, B, F* will be directly linked to expression of rRNA and growth rate, but they would be expressed at a lower level than the rRNA, which are among the most highly transcribed genes in the cell. We point out that the dual terminators of ribosomal operon B, including the *rrB* gene, are among the most highly used transcription terminator sequences on plasmids for cloning and protein expression (Denèfle et al., 1987; Andrews et al., 1996; Rogers et al., 2015; Wille et al., 2015; Engstrom and Pfleger, 2017). Our demonstration that the RrA, B, F molecules can interact with at least two pleiotropic regulatory proteins in *E. coli* (Hfq and CsrA), warrants careful consideration of whether expression of such putative sRNAs from a high copy number plasmid is desirable for a given application.

Several studies on eukaryotic organisms have identified potential miRNAs transcribed as part of rRNA primary transcripts (Wei et al., 2013; Chak et al., 2015; Asha and Soniya, 2017), and the murine-specific miR-712, transcribed as a part of a spacer element in the pre-rRNA transcript, has been shown to be involved in endothelial inflammation and atherosclerosis (Son et al., 2013). To our knowledge, the RrA, B, F sRNAs described here represent the first report of sRNAs transcribed as part of rRNA primary transcripts in a prokaryote. In conclusion, three out of seven rRNA operons in *E. coli* contains an arrangement of dual terminators with small defined RNAs encoded in between, and these RNAs are conserved both in structure and sequence among species belonging to different genera of Enterobacteriales. We have found that these RNAs interact with both Hfq and CsrA but has been unable to demonstrate clear phenotypes of both deletion and overexpression mutants. We presume that the conservation among species indicate a function for the RNAs but that this function is still not fully understood.

## Data Availability Statement

The datasets presented in this study can be found in online repositories. The names of the repository/repositories and accession number(s) can be found below: http://www.ebi.ac.uk/pride/archive/projects/PXD013749.

## Author Contributions

TS, MK, SS, and MS wrote the manuscript, conceived and designed the research. TS, MK, BK, EH, SS, and MS designed the experiments. MK (Hfq) and TS (CsrA) performed the experiments. All authors contributed to the article and approved the submitted version.

## Conflict of Interest

The authors declare that the research was conducted in the absence of any commercial or financial relationships that could be construed as a potential conflict of interest.

## Footnotes

1 www.ecogene.org

2 https://blast.ncbi.nlm.nih.gov/Blast.cgi

3 http://unafold.rna.albany.edu/?q=mfold
