# Supplementary material for: Three Ribosomal Operons of Escherichia coli Contain Genes Encoding Small RNAs That Interact With Hfq and CsrA in vitro
Source: Front Microbiol. 2021 May 11;12:625585. doi: 10.3389/fmicb.2021.625585 (PMC8144298; doi:10.3389/fmicb.2021.625585)
Supplement: Supplementary file 1 [file Data_Sheet_1.PDF]

Supplementary Material

1 Supplementary Figures

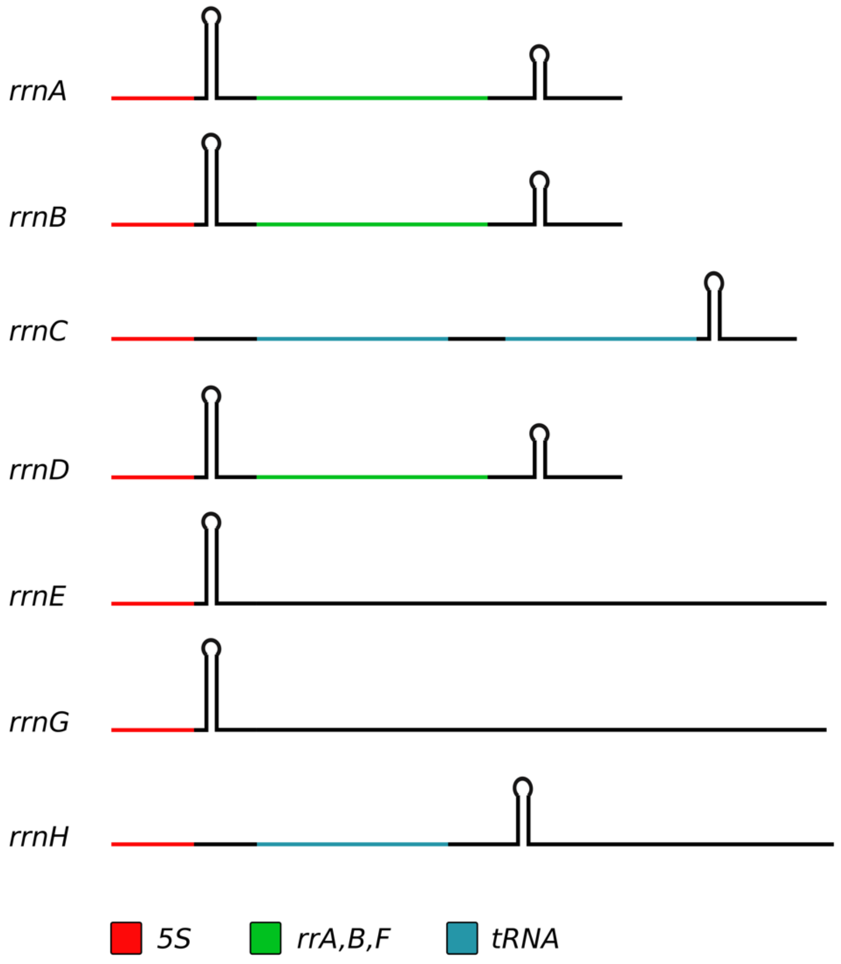

Figure S1: Schematic of the terminator area of all seven ribosomal RNA operons from *E. coli.* The figure illustrates the sequences downstream from the 5S genes for each of the rRNA operons (the *rrnD* operon contains two 5S genes, here only the terminal one is presented). The 3'-end of the 5S gene is shown in red, the annotated *rrA, B, F* sequences (corresponding to the sequence between the asterisks in Figure 1) in green and any tRNA genes in blue. Terminator sequences are shown by stem loop structures. The terminators are drawn based on prediction from (Lesnik et al., 2001).

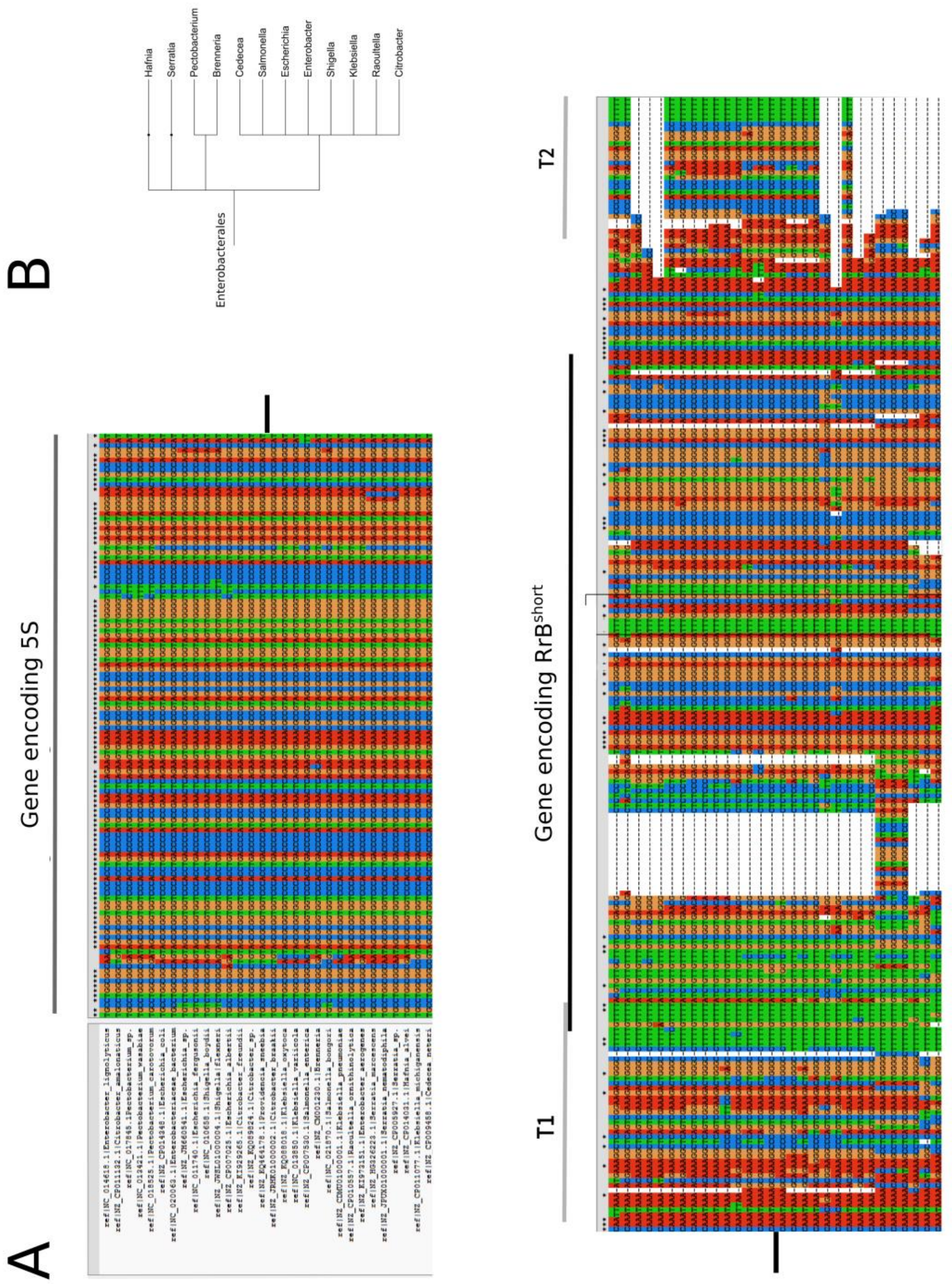

Figure S2: ClustalX alignment of the sequences found in a blast search for homology to the 5S-**T1-RrB-T2 sequence**. **(A)** ClustalW alignment of the 30 sequences used as input for the structural conservation analysis (Fig. 5 of the main text). These sequences were found in a BLAST search to be homologous to the 5S-T1-RrB-T2 sequence of *E. coli*, see supplementary text. All the presented sequences come from the order of Enterobacteriales and represent 12 different genera. **(B)** Phylogenetic three showing the evolutionary relationship of the 12 different genera used in the analysis.

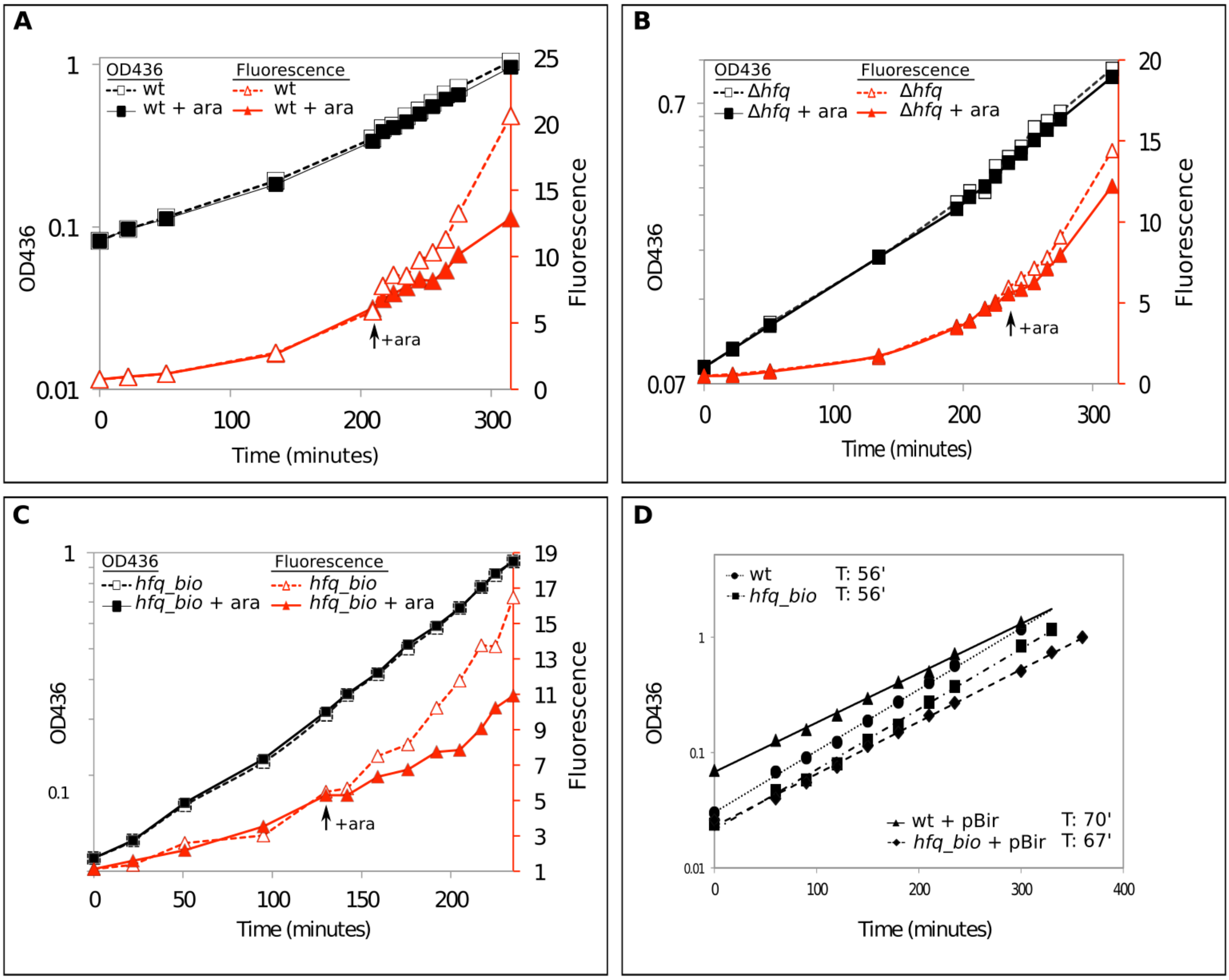

Figure S3: Functionality of the epitope-tagged Hfq (Hfq_bio). Induction of the Hfq-dependent sRNA Qrr2 represses HapR-GFP expression. Panels **(A)**, **(B)** and **(C)** show growth as optical density (OD_{436}, left axis) and HapR-GFP expression as fluorescence (arbitrary units, right axis). Expression of Qrr2 from the pQrr2 plasmid was induced by addition of arabinose (0.2%), at the points indicated by the arrows (+ara) **(A)** wt (MG1655 pQrr2 and pHapR-GFP) **(B)** Δ*hfq* (Δ*hfq* pQrr2 and pHapR-GFP). The regulation of HapR-GFP by Qrr2 is abolished in a Δ*hfq* strain. **(C)** Hfq_bio (Hfq_bio pQrr2 and pHapR-GFP). The Hfq_bio is able facilitate the Qrr2 mediated regulation of HapR-GFP similar to the wt Hfq. **(D)** comparison of growth rates of strains used for Hfq-tag experiments. wt (MG1655), Hfq_bio, wt + pBirA and Hfq_bio + pBirA, grown in MOPS supplemented with 0.2% glucose.The pQrr2 and pHapR-GFP constructs were used to show the functionality of Hfq_bio because they were already at hand in our laboratory and have previously been used in *E. coli* as an expression platform to study the Hfq-dependent Qrr sRNAs(Bardill et al., 2011; Hansen et al., 2015).

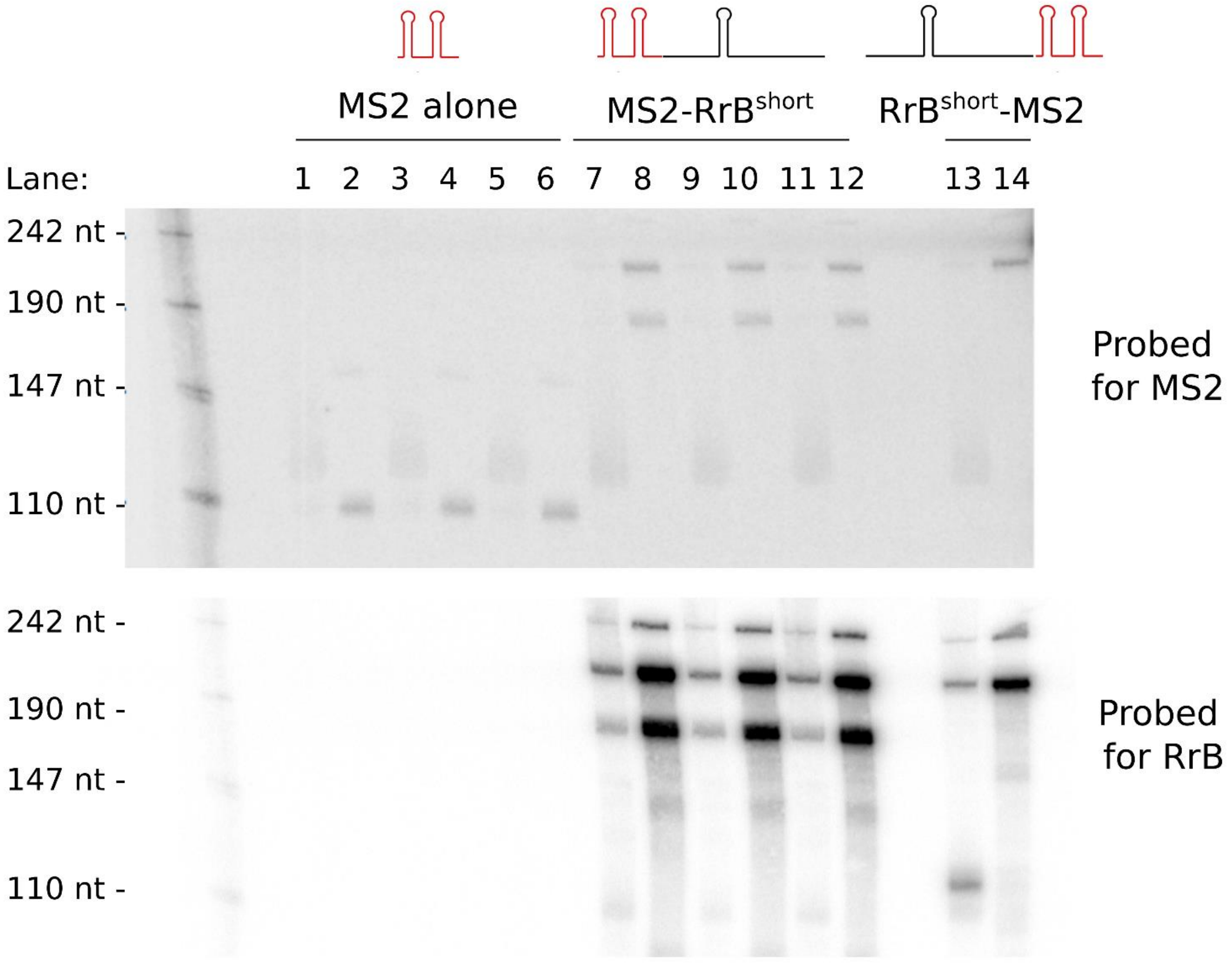

Figure S4: Northern blot of affinity-purified MS2-tagged RrB^{short} RNA. Affinity purification of MS2 alone (lanes 1-6) and RrB^{short} tagged with MS2 at the 5'-end (MS2-RrB^{short}, lanes 7-12) was done in triplicates while RrB^{short} tagged at the 3'-end was done as a single experiment (RrB^{short}–MS2, lanes 13, 14). Each sample is represented by two lanes on the Northern blot: the left one being total RNA from cell lysate and the right one being the affinity-purified RNA. The same blot was probed for the MS2 sequence and the RrB sequence as indicated on the figure (see table S4 for probe sequences).

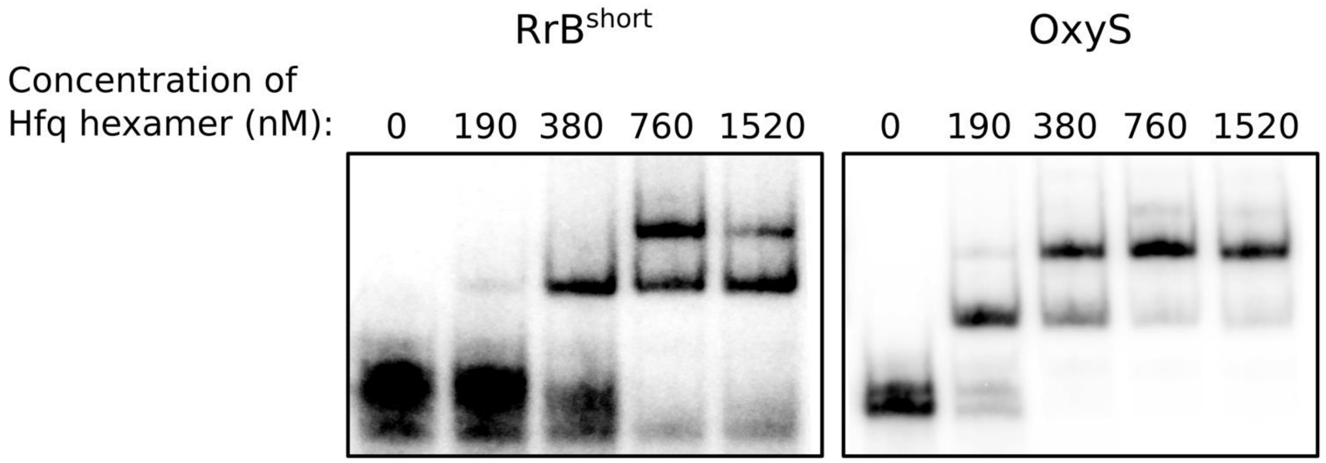

Figure S5: Electrophoretic mobility shift assays (EMSA) of RrB^{short} and OxyS with Hfq. In a total volume of 10 ul, 40 fmol ^{32}P-5'-end labeled transcript, 2 pmol unlabeled random RNA oligo 75 nt long (TAG-Copenhagen), was incubated with or without Hfq in buffer; 100 mM KCl, 1 mM DTT, 20 mM HEPES pH 8. The samples were incubated 20 minutes at 37 $^{\circ}$C, transferred to ice for 10 minutes and subsequently separated on a 5 % non-denaturing poly acrylamide gel at 4 $^{\circ}$C.

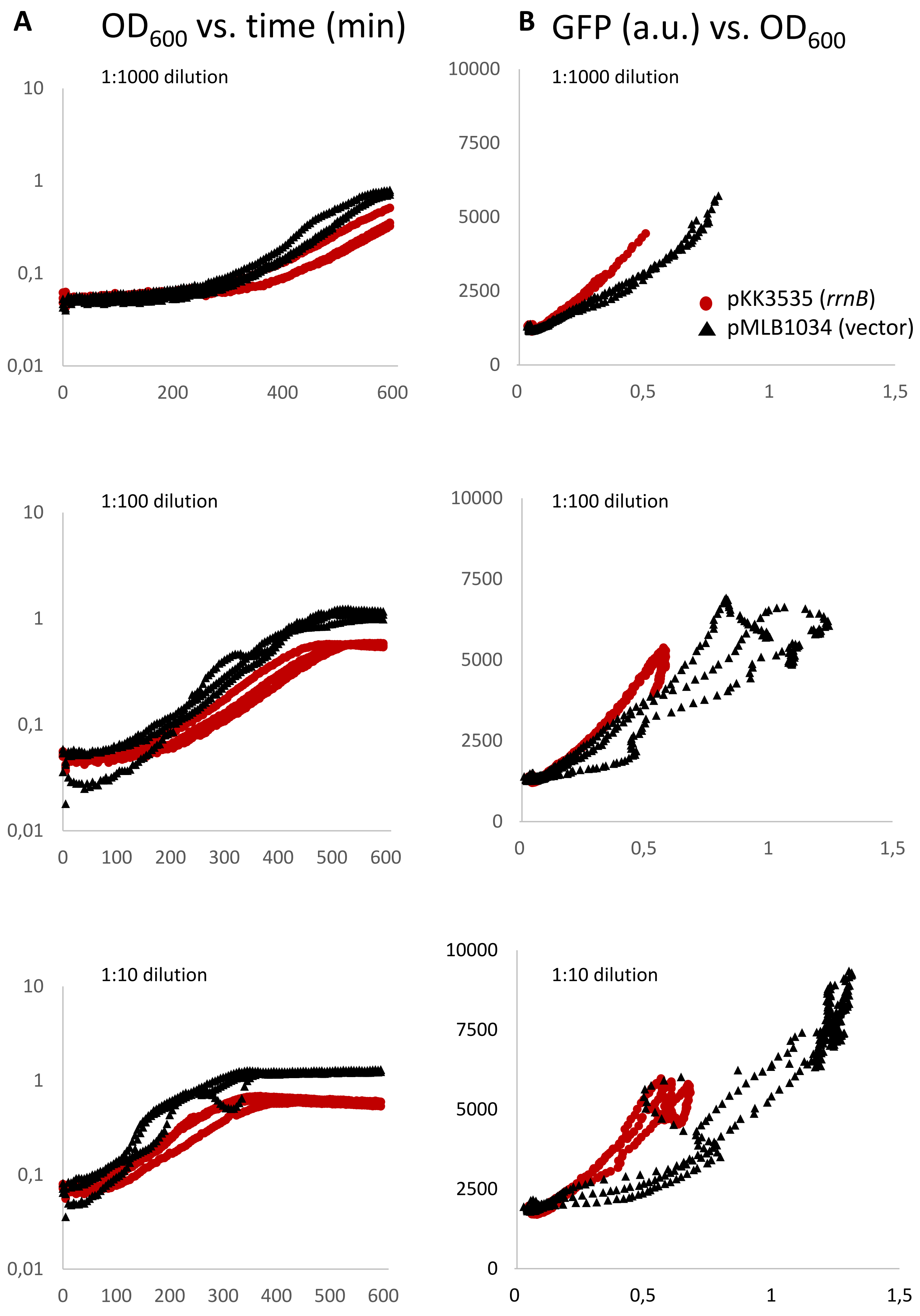
A
OD600 vs. time (min)
B
GFP (a.u.) vs. OD600
1:1000 dilution
1:100 dilution
1:10 dilution
pKK3535 (rrnB)
pMLB1034 (vector)
10
1
0,1
0,01
0
100
200
300
400
500
600
10000
7500
5000
2500
0,5
1,5

C

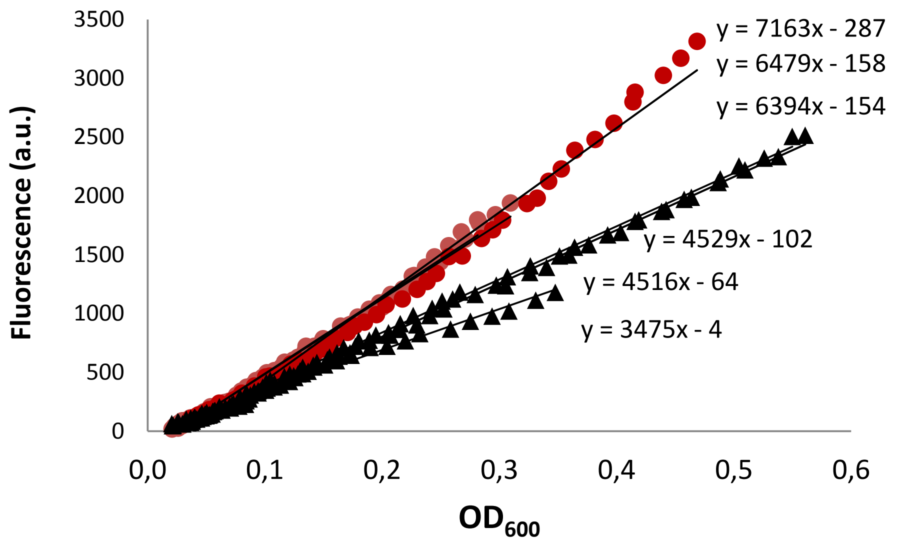

Figure S6: Fluorescence from *glgC-gfp* protein fusion increased upon overexpression of the *rrnB* operon from plasmid pKK3535, which includes the *rrB* gene. Overnight cultures of the Δ*rrA*, *B*, *F* strain carrying a plasmid-borne translational *glgC-gfp* fusion (pTSS16) as well as either pKK3535 containing the *rrnB* operon (red circles), or the control plasmid pMLB1034 (black triangles), were monitored for GFP fluorescence and absorbance (OD_{600}) during 6 hours of incubation in a microtiter plate reader with shaking. To capture both the exponential and stationary phases of growth, overnight cultures grown in MOPS medium supplemented with 0.2% glucose, 100 µg/ml ampicillin and 10 µg/ml chloramphenicol were diluted 1:1000 (top panels), 1:100 (middle panels) or 1:10 (bottom panels) into fresh medium of the same composition. (**A)** growth curves obtained by absorbance readings (OD_{600}) in an Omega Fluostar plate reader (BMG). (**B)** Green fluorescence as a function of OD is shown for three independent cultures of each type. (a.u.) indicates arbitrary units of fluorescence. (**C)** GFP production rates increased ~1.6-fold in the presence of RrB during exponential growth, as calculated from the slopes of the linear portions of the graphs shown in the top panel of Figure S5B.

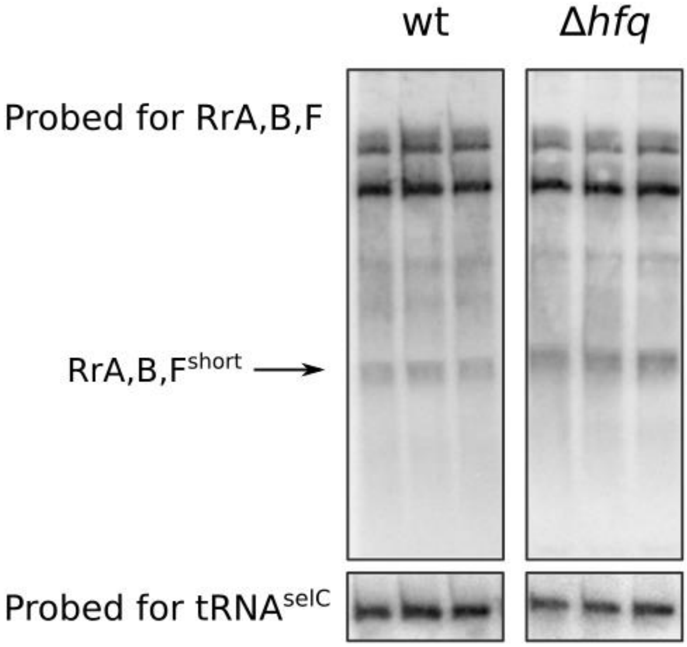

Figure S7: Northern blot of total RNA from wildtype and an *hfq* mutant. Northern blot made from total RNA harvested from cultures of MG1655 and an isogenic Δ*hfq* strain. The cultures were grown in MOPS minimal media supplemented with 0.2% glucose, 10 mg/l uracil. Right before RNA purification, spike-in cells overexpressing the tRNA gene *selC* were added to each experimental sample (see Materials and Methods). 8-10 µg RNA was loaded in each lane. The blot was probed for RrA, B, F and $tRNA^{selC}$ as indicated. Three technical replicates were prepared for each culture.

2 Supplementary Text for Fig. 5 and Fig. S2

The structure and sequence of RrA, B, F^{short} is conserved among many members of the Enterobacteriaceae. To determine the degree of conservation of the sequence and genomic location of *rrA, B, F* in other species, we performed a BLAST homology search(Coordinators, 2016) using the *E. coli* MG1655 sequence encoding *rrfB*, T1, *rrB* and T2 (Fig.1 and S2). Inclusion of the highly conserved *rrfB* sequence was necessary to avoid too much noise due to the repetitive nature of the T1-*rrB*-T2 sequences in the NCBI database. The hits selected from the search contained homology from the start of *rrfB* to the end of the annotated *rrB*(Zhou and Rudd, 2012) and only the hit with the highest homology score from each species was selected.
The selected 31 hits can be sub-divided into 3 groups based on the 16S rRNA homology(Pruesse et al., 2007); the closely related: *Salmonella, Shigella, Klebsiella, Escherichia, Pectobacterium, Brenneria*; the more distant: *Citrobacter, Enterobacter, Provedencia, Raoultella*; and the most distant: *Cedecea, Serratia* and *Hafnia*. The location of the T2 terminator downstream of the *rrB* is conserved in 18 species from the genera; *Salmonella, Shigella, Klebsiella, Enterobacter, Citrobacter, Provendencia* and in one of the species from the genus of *Serratia* (Fig. S2A).
Having found this homology, we compared sequence versus structure conservation of RrB^{short}. For that purpose we used the locARNA consensus structure prediction algorithm(Schmiedl et al., 2012; Will et al., 2012; Amit et al., 2014), and since 30 sequences is the maximum for locARNA, the most distant species, *Serratia plymuthia*, was left out of the analysis. Figure 5 shows the locARNA consensus structure for the 30 species.
When we used the locARNA algorithm on the RrB^{short} sequence it led to the structure shown in Figure 5, retaining the stem 2, 3, and 4 and thus displaying almost the same fold as the experimentally determined structure found in Fig 6.

3 Supplementary Methods - Mass Spectrometry

3.1 Gel electrophoresis

Proteins in NuPAGE® LDS sample buffer (Life Technologies) were reduced with 50 mM DTT at 70 °C for 10 minutes and alkylated with 120 mM Iodoacetamide at room temperature for 20 minutes and separated on NuPAGE® Novex® 4-12 % Bis-Tris gels (Life Technologies) with MOPS buffer according to manufacturer’s instructions. Gels were washed three times for 5 min with water and stained for 45 min with Simply Blue™ Safe Stain (Life Technologies). After washing with water for 2 h, each gel lane was cut into 13 slices.

3.2 In-Gel Digestion

The excised gel bands were destained with 30 % acetonitrile in 0.1 M NH_4HCO_3 (pH 8), shrunk with 100 % acetonitrile, and dried in a vacuum concentrator (Concentrator 5301, Eppendorf, Germany). Digests were performed with 0.1 µg trypsin per gel band overnight at 37 °C in 0.1 M NH_4HCO_3 (pH 8). After removing the supernatant, peptides were extracted from the gel slices with 5 % formic acid, and extracted peptides were pooled with the supernatant.

3.3 NanoLC-MS/MS Analysis

NanoLC-MS/MS analyses were performed on an Orbitrap Fusion (Thermo Scientific) equipped with an EASY-Spray Ion Source and coupled to an EASY-nLC 1000 (Thermo Scientific). Peptides were loaded on a trapping column (2 cm x 75 µm ID, PepMap C18, 3 µm particles, 100 Å pore size) and separated on an EASY-Spray column (25 cm x 75 µm ID, PepMap C18, 2 µm particles, 100 Å pore size) with a 30-minute linear gradient from 3% to 30% acetonitrile and 0.1% formic acid.

Both MS and MS/MS scans were acquired in the Orbitrap analyzer with a resolution of 60,000 for MS scans and 15,000 for MS/MS scans. HCD fragmentation with 35 % normalized collision energy was applied. A Top Speed data-dependent MS/MS method with a fixed cycle time of 3 seconds was used. Dynamic exclusion was applied with a repeat count of 1 and an exclusion duration of 30 seconds; singly charged precursors were excluded from selection. Minimum signal threshold for precursor selection was set to 50,000. Predictive AGC was used with AGC a target value of 2e5 for MS scans and 5e4 for MS/MS scans. EASY-IC was used for internal calibration.

3.4 MS data analysis

Raw MS data files were analyzed with MaxQuant version 1.5.3.30 (Cox and Mann, 2008). Database search was performed with Andromeda, which is integrated in the utilized version of MaxQuant. The search was performed against the UniProt E. coli database or Salmonella database. Additionally, a database containing common contaminants was used. The search was performed with tryptic cleavage specificity with 3 allowed miscleavages.
Protein identification was under control of the false-discovery rate (<1% FDR on protein and peptide level). In addition to MaxQuant default settings, the search was performed against following variable modifications: Protein N-terminal acetylation, Gln to pyro-Glu formation (N-term. Gln) and oxidation (Met). Carbamidomethyl (Cys) was set as fixed modification. For protein quantitation, the LFQ intensities were used(Cox et al., 2014). Proteins with less than two identified razor/unique peptides were dismissed.

Further data analysis was performed using R scripts developed in-house. For discrimination of unspecifically enriched proteins, LFQ intensities were quantile normalized, and median intensities were calculated. Missing LFQ intensities in the control samples were imputed with values close to the baseline. Data imputation was performed with values from a standard normal distribution with a mean of the 5% quantile of the combined log10-transformed LFQ intensities and a standard deviation of 0.1. For the identification of significantly enriched proteins, boxplot outliers were identified in intensity bins of at least 300 proteins. Log2 transformed protein ratios of sample versus control with values outside a 1.5x (potential) or 3x (extreme) interquartile range (IQR), respectively, were considered as significantly enriched.

4 Strain list

Name	Description	Reference
wt (MAS1081)	MG1655; *rph*+ *gatC*+ *glpR*+ Genome sequenced	Svenningsen et al. 2017
Δ*rrA, B, F* (TSS80)	MAS1081; Δ(*rrA*) : Δ(4038719-4038825)::*valZlysY(780240-780475)*, Δ(*rrB*) : Δ(4169839-4169942), Δ(*rrF*) : Δ(3421385-3421282)::*tyrT(1286876-1286735)*	This work
csrA::kan (TSS244)	MAS1081; *csrA::kan* allele from (Romeo et al., 1993)	This work
hfq_bio	MG1655; Hfq with the following amino acid sequence; GLNDIFEAQKIEWH2 at the C-terminal (biotinylation sequence)	This work
Δ22TLRs (TSS107)	MAS1081; Δ22 TLR: Δ (*lysW/Y/Z/Q, valT/Z*, rtTWYZQ) : Δ(779872-781031), Δ(*tyrV*, *rtV*1-3, *rtT*) : Δ(1285791-1286991), Δ(*rsW*') : Δ(925059-925098), Δ(*rsX*1- 4) : Δ(1096182-1096779), Δ(*argY/Z/Q, raQ/Z/V*) : Δ(2815672-2816445), Δ(*rrA*) : Δ(4038719-4038825)::*valZlysY(780240-780475)*, Δ(*rrB*) : Δ(4169839-4169942), Δ(*rrF*) : Δ(3421385-3421282)::*tyrT(1286876-1286735)*, Δ(*rpK*) : Δ(3706471-3706638), Δ(*rlV*) : Δ(2519426-2591521)	This work
MG1655	*rph1*	Lab. collection
hfq-FLAG (JVS-814)	W3110; 1xFLAG-*hfq*	Vogel lab. collection
CsrA-3xFLAG	MG1655, *csrA*::3xFLAG::kan	(Jorgensen et al., 2013)
Δ*hfq*	MG1655; Δ(*hfq) : Δ*(4398291-4398651)	This work
Δ*lacZ*	MAS1081: Δ*lacZYA::cat*	Lab. collection

Table S1: List of strains used in this study. Genomic positions refer to the *E. coli* genome U00096.2

5 Plasmid list

Name	Description	Reference
pRrfB-RrB (pTSS1)	pJFR1 with the rrBDownPst#1-rrBUpBamHI#1 PCR fragment replacing the small PstI-BamHI fragment of the plasmid. Plasmid expressing *rrfB* (5S) and *rrB* from P_{lac}	This work
pRrB (pTSS2)	pJFR1 with the rrBDownPst#1-rrBUpBamHI#2 PCR fragment replacing the small PstI-BamHI fragment of the plasmid. Plasmid expressing a truncated *rrfB* (5S) and *rrB* from P_{lac}	This work
pJFR1	pUC18(Yanisch-Perron et al., 1985) modified at EcoRI site to contain a stop codon at the 6th codon in the *lacZα* reading frame.	Fricke, J (unpublished)
pMS2-RrB (pTSS4)	pNS21 with the rrBclon#1XbaIfrwd-rrBclon#1XbaIrev PCR fragment inserted in the XbaI site. The orientation of the insert was with the 5'-end of *rrB* closest to the MS2 sequence. This was verified by sequencing. Plasmid expressing RrB^{short} 5'-tagged with the MS2*-sequence	This work
pRrB-MS2 (pTSS6)	pNS21 with the rrBclon#1NheIfrwd-rrBclon#1NheIrev PCR fragment inserted in the NheI site. The orientation of the insert was with the 3'-end of *rrB*^{short} closest to the MS2 sequence. This was verified by sequencing. Plasmid expressing RrB^{short} 3'-tagged with the MS2*-sequence	This work
pNS21	pZE12-luc with modified p15A origin, MS2*-aptamer sequence and VrrA terminator sequence.	(Said et al., 2009)
pGlgC-GFP (pTSS16)	pXG10-SF with the TSSO-21-TSSO-22 PCR fragment replacing the small NsiI-NheI site. *glgC*-sf-*gfp* translational fusion.	This work
pPgaA-lacZ (pTSS36)	pTSS35 with the TSSO-91-TSSO-92 PCR fragment	This work

	replacing the small NheI-BamHI fragment. *pgaA-lacZ* translational fusion.	
pBirA	Plasmid derived from pACYC184, expressing *birA* from a p_{lac} promoter	Avidity
pHapR-GFP	pSLS73: pLAFR3 cosmid containing a *hapR-gfp* translational fusion	(Svenningsen et al., 2008)
pQrr2	Plasmid expressing the *qrr*2 gene from *Vibrio cholerae* from pBAD promoter	(Hansen et al., 2015)
pCsrB (pTSS13)	pIDA1 with the TSSO-8-TSSO-9 PCR fragment replacing the small EcoRI-NcoI fragment. *csrB* expression plasmid.	This work
pXG0	pZA31-luc with a pSC101 replicon.	(Urban and Vogel, 2009)
pXG10-SF	Expressing sf-*gfp* from a P_{LtetO} promoter.	(Corcoran et al., 2012)
pGH253	mini-R1 plasmid with *lacZYA,* no promoter. *ts.*	Hann, G (PhD Thesis 2017 Gerdes lab)
pTSS35	pGH253 with the TSSO-89-TSSO-90 PCR fragment replacing the small EcoRI-BamHI fragment. *lacZ* expressed from P_{lacI}	This work
pBAD-RBS-csrA::3xFLAG	csrA3XFLAG with optimized ribosome binding site for increased expression, expressed from P_{BAD}.	(Jorgensen et al., 2013)
pIDA1	The *bla* and the low copy p15A origin from pACYC177(Chang and Cohen, 1978) on a HinDIII-ClaI fragment + the multi-cloning site from pBAD24(Guzman et al., 1995) from HinDIII-EcoRI + a PCR amplified fragment of a Ptac *lacI*$^+$ fragment from pCYB2 (New England Biolabs) on an EcoRI-ClaI fragment. Sequences cloned into the multi-cloning site are expressed from P_{tac} upon IPTG induction.	Muñoz-Gómez, A. J. & Sjøgaard, I. M. Z. (unpublished)
pMazF (pMCD3326)	pBAD33; BAD::SD_{opt}::*mazF*	(Christensen-Dalsgaard and Gerdes, 2008)
pKK3535	pBR322-based plasmid containing the *rrnB* operon	(Brosius et al., 1981)
pMLB1034	pBR322-derived vector plasmid containing a promoterless lacZ gene. Used here as control vector for comparison with pKK3535	(Shapira et al., 1983)
pRrB-3GGA	pUC57 with a mutant version of *rrB* that has three GGA motifs each changed to "TTT".	This work

Table S2: List of plasmids used in this study

6 DNA oligos:

6.1 Oligos used as primers:

Name	Sequence	Description	Use
rrBDownPst#1	agatactgcagCAGGAAGAGTTTGTAGAAAC	Antisense to downstream area of *rrB*, including a PstI restriction site and 5 nt overhang	Construction of pTSS1/2
rrBUpBamHI#1	gattaggatccAACGCAGAAGCGGTCTGATA	Sense to middle part of *rrnB*, including a BamHI restriction site and 5 nt overhang	Construction of pTSS1
rrBUpBamHI#	cacctggatccATGCCGAACT	Sense to beginning of *rrnB*.	Construction of

2	CAGAAGTGAA	Including a BamHI restriction site and a 5 nt overhang	pTSS2
rrBclon#1XbaI frwd	tctatctagaTTTTATCTGTTGTTTGTCGGTGAAC	Sense to 5'-end of *rrB*^{short}. Including a XbaI restriction site and a 4 nt overhang	Construction of pTSS4
rrBclon#1XbaI rev	tatgtctagaTTATGGCGGGCGTCCTG	Antisense to 3'-end of *rrB*^{short}. Including a XbaI restriction site and a 4 nt overhang	Construction of pTSS4
rrBclon#1NheI frwd	tagagctagcTTATGGCGGGCGTCCTG	Sense to 5'-end of *rrB*^{short}. Including a NheI restriction site and a 4 nt overhang	Construction of pTSS6
rrBclon#1NheI rev	tctagctagcTTTTATCTGTTGTTTGTCGGTGAAC	Antisense to 3'-end of *rrB*^{short}. Including a NheI restriction site and a 4 nt overhang	Construction of pTSS6
TSSO-9	ttctgaattcGTCGACAGGGAGTCAGACAA	Sense to 5'-end of *csrB*. Including a EcoRI restriction site and 4 nt overhang	Construction of pTSS13
TSSO-10	ggatccatggAATAAAAAAAGGGAGCACTGTATTCAC	Antisense to 3'-end of *csrB*. Including a NcoI restriction site and 4 nt overhang	Construction of pTSS13
TSSO-21	gttttatgcatTTTTACCTGCTGGGGAGTGG	Sense to 5'-UTR of *glgC*. Including a NsiI restriction site and 5 nt overhang	Construction of pTSS16
TSSO-22	gttttgctagcTAAGTGATCGTTCTTCTCTAAACTAACCAT	Antisense to start of *glgC* open reading frame. Including a NheI restriction site and 5 nt overhang	Construction of pTSS16
cRACE rrB-2 1F	CCGGGAGCGGATTTGAACGT	Sense to *rrB*	First round PCR amplification in cRACE
cRACE rrB-2 1R	CGCTCCCGGCGGATTTGT	Antisense to *rrB*	First round PCR amplification in cRACE
cRACE rrB-2 2F	gtctcgtgggctcggagatgtgtataagagacagGAACGTTGCGAAGCAACGGC	Nested primer, sense to *rrB.* Including adapter and index sequences for sequencing on the Illumina platform	Second round PCR amplification in cRACE.
cRACE rrB-2 2R	tcgtcggcagcgtcagatgtgtataagagacagTTTGTCCTACTCAGGAGAGCGT	Nested primer, antisense to *rrB.* Including adapter and index sequences for sequencing on the Illumina platform	Second round PCR amplification in cRACE.
TSSO-91	gttttgctagcAGGCATTGGGATTTATGCCGTA	Sense to 5'-UTR of *pgaA.* Including a NheI restriction site and 5 nt overhang	Construction of pTSS36
TSSO-92	gttttggatccCGGGCACCTTTTTCTGCTAC	Antisense to beginning of the *pgaA* open reading frame. Including a BamHI site and 5 nt overhang	Construction of pTSS36
OxyS FW inc T7	TAATACGACTCACTATAGGgaaacggagcggcacctcttttaac	Sense to 5'-end of *oxyS*. Including a T7 promoter sequence	*In vitro* transcription of *oxyS*
OxyS RW	AGCGGATCCTGGAGATCCGC	Antisense to 3'-end of *oxyS*	*In vitro* transcription of *oxyS*

TSSO-77	TAATACGACTCACTATAgGTC GACAGGGAGTCAGACAA	Sense to 5'-end of *csrB*. Including T7 promoter sequence and and an additional "G"	*In vitro* transcription of *csrB*
TSSO-78	AATAAAAAAAGGGAGCACTGT ATTCAC	Antisense to 3'-end of *csrB*	*In vitro* transcription of *csrB*
TSSO-79	TAATACGACTCACTATAgGTT TTATCTGTTGTTTGTCGGTGA AC	Sense to 5'-end of *rrA, B, F*^{short}. Including a T7 promoter sequence and an additional "G"	*In vitro* transcription of *rrA, B, F*^{short} and *rrA, B, F*^{short}-3GGA
TSSO-80	TTATGGCGGGCGTCCT	Antisense to 3'-end of *rrA, B, F*^{short}	In *vitro* transcription of *rrA, B, F*^{short}
EHO-1722	TTATGGCGGGCGAAATGC	Antisense to 3'-end of *rrA, B, F*^{short}	In *vitro* transcription of *rrA, B, F*^{short}-3GGA
TSSO-89	gttttgaattcGGCGAAGCGG CATGCAT	Sense to *lacI* promoter. Including a EcoRI restriction site and 5 nt overhang	Construction of pTSS35
TSSO-90	gttttggatccgttttgctag cCACCACCCTGAATTGACTCT CTT	Antisense to *lacI* promoter. Including BamHI and NheI restrictions sites and 5 nt overhang	Construction of pTSS35
ClaIpCYB2lac IpromoterUP	acaccatcgatTGGTGCAAAA CCTTTC	Sense to promoter of *lacI.* Including a ClaI restriction site and a 5 nt overhang	Construction of pIDA1
EcoRIpCYB2T ACpromoter DOWN	ttggtgaattcCTCGTGAAAA CACCTAAAC	Antisense to promoter of P_{tac}. Including a EcoRI restriction site and a 5 nt overhang	Construction of pIDA1

Table S3: List of oligos used as primers in this study. All nucleotide sequences are listed in the 5'- to 3'- direction.

6.2 Oligos used as probes:

Name	Sequence
RrABF	GGATTTGTCCTACTCAGGAG
5S	ACACTACCATCGGCGCTAC
$tRNA^{his}$	CACGACAACTGGAATCACAATCC
$tRNA^{glu}$	CCTGTTACCGCCGTGAAAGGG
OxyS	GAGATCCGCAAAAGTTCACGTT
rrB68-8 upstrm	CAGATAAAACGAAAGGCCCAGTCTTTCGACTGAGCCTTTCGTTTTATTTGATGC CTGGCA
rrB45-15	GGAGAGCGTTCACCGACAAACAACAGATAAAACGAAAGGCCCAGTCTTTCGACT GAGCCT
rrB10-50	GTTCAAATCCGCTCCCGGCGGATTTGTCCTACTCAGGAGAGCGTTCACCGACAA ACAACA
rrB3'map53-10	ATTTGATGCCTGGCAGTTTATGGCGGGCGTCCTGCCCGCCACCCTCCGGGCCGT TGCTTCGCA
rrB3'-map40-20	CTTCTGCTTAATTTGATGCCTGGCAGTTTATGGCGGGCGTCCTGCCCGCCACCC TCCGGG

rrB3'-map30-35	ATCCGTCAGGATGGCCTTCTGCTTAATTTGATGCCTGGCAGTTTATGGCGGGCG TCCTGCCCGCC
rrB54-8 upstrm	CAGATAAAACGAAAGGCCCAGTCTTTCGACTGAGCCTTTCGTTTTA
rrB50-8 upstrm	AGATAAAACGAAAGGCCCAGTCTTTCGACTGAGCCTTTCGT
MS2 (JVO-3562)(Said et al., 2009)	GTGTCTGAAAAACGTACCCTGAT

Table S4: List of oligos used as probes in this study. All nucleotide sequences are listed in the 5'- to 3'- direction.

7 Supplementary References

Amit, M., Backofen, R., Heyne, S., Landau, G. M., Mohl, M., Otto, C., et al. (2014). Local Exact Pattern Matching for Non-Fixed RNA Structures. *IEEE/ACM Trans. Comput. Biol. Bioinforma.* 11, 219–230. doi:10.1109/TCBB.2013.2297113.

Bardill, J. P., Zhao, X., and Hammer, B. K. (2011). The Vibrio cholerae quorum sensing response is mediated by Hfq-dependent sRNA/mRNA base pairing interactions. *Mol. Microbiol.* 80, 1381–1394. doi:10.1111/j.1365-2958.2011.07655.x.

Brosius, J., Ullrich, A., Raker, M. A., Gray, A., Dull, T. J., Gutell, R. R., et al. (1981). Construction and fine mapping of recombinant plasmids containing the rrnB ribosomal RNA operon of E. coli. *Plasmid* 6, 112–118. doi:10.1016/0147-619x(81)90058-5.

Chang, A. C., and Cohen, S. N. (1978). Construction and characterization of amplifiable multicopy DNA cloning vehicles derived from the P15A cryptic miniplasmid. *J. Bacteriol.* 134, 1141 LP – 1156.

Christensen-Dalsgaard, M., and Gerdes, K. (2008). Translation affects YoeB and MazF messenger RNA interferase activities by different mechanisms. *Nucleic Acids Res.* 36, 6472–6481. doi:10.1093/nar/gkn667.

Coordinators, N. R. (2016). Database resources of the National Center for Biotechnology Information [ftp://ftp.ncbi.nih.gov/genomes/Bacteria]. *Nucleic Acids Res.* 44, D7–D19. doi:10.1093/nar/gkv1290.

Corcoran, C. P., Podkaminski, D., Papenfort, K., Urban, J. H., Hinton, J. C. D., and Vogel, J. (2012). Superfolder GFP reporters validate diverse new mRNA targets of the classic porin regulator, MicF RNA. *Mol. Microbiol.* 84, 428–445. doi:10.1111/j.1365-2958.2012.08031.x.

Cox, J., Hein, M. Y., Luber, C. A., Paron, I., Nagaraj, N., and Mann, M. (2014). Accurate Proteome-wide Label-free Quantification by Delayed Normalization and Maximal Peptide Ratio Extraction, Termed MaxLFQ. *Mol. Cell. Proteomics* 13, 2513–2526. doi:10.1074/mcp.M113.031591.

Cox, J., and Mann, M. (2008). MaxQuant enables high peptide identification rates, individualized p.p.b.-range mass accuracies and proteome-wide protein quantification. *Nat. Biotechnol.* 26, 1367.

Guzman, L. M., Belin, D., Carson, M. J., and Beckwith, J. (1995). Tight regulation, modulation, and high-level expression by vectors containing the arabinose PBAD promoter. *J. Bacteriol.* 177, 4121–4130. doi:10.1128/jb.177.14.4121-4130.1995.

Hansen, S., Krishna, S., Semsey, S., and Lo Svenningsen, S. (2015). Effects of Four Different

Regulatory Mechanisms on the Dynamics of Gene Regulatory Cascades. *Sci. Rep.* 5, 12186. doi:10.1038/srep12186.

Jorgensen, M. G., Thomason, M. K., Havelund, J., Valentin-Hansen, P., and Storz, G. (2013). Dual function of the McaS small RNA in controlling biofilm formation. *Genes Dev.* 27, 1132–1145. doi:10.1101/gad.214734.113.

Lesnik, E. A., Sampath, R., Levene, H. B., Henderson, T. J., McNeil, J. A., and Ecker, D. J. (2001). Prediction of rho-independent transcriptional terminators in Escherichia coli. *Nucleic Acids Res.* 29, 3583–94. doi:10.1093/nar/29.17.3583.

Pruesse, E., Quast, C., Knittel, K., Fuchs, B. M., Ludwig, W., Peplies, J., et al. (2007). SILVA: a comprehensive online resource for quality checked and aligned ribosomal RNA sequence data compatible with ARB. *Nucleic Acids Res.* 35, 7188–7196. doi:10.1093/nar/gkm864.

Romeo, T., Gong, M., Liu, M. Y., and Brun-Zinkernagel, A.-M. (1993). Identification and Molecular Characterization of *csrA*, a Pleiotropic Gene from *Escherichia coli* That Affects Glycogen Biosynthesis, Gluconeogenesis, Cell Size, and Surface Properties. *J. Bacteriol.* 175, 4744–55. doi:10.1128/JB.175.15.4744-4755.1993.

Said, N., Rieder, R., Hurwitz, R., Deckert, J., Urlaub, H., and Vogel, J. (2009). In vivo expression and purification of aptamer-tagged small RNA regulators. *Nucleic Acids Res.* 37. doi:10.1093/nar/gkp719.

Schmiedl, C., Möhl, M., Heyne, S., Amit, M., Landau, G. M., Will, S., et al. (2012). “Exact Pattern Matching for RNA Structure Ensembles,” in, 245–260. doi:10.1007/978-3-642-29627-7_27.

Shapira, S. K., Chou, J., Richaud, F. V, and Casadaban, M. J. (1983). New versatile plasmid vectors for expression of hybrid proteins coded by a cloned gene fused to lacA gene sequences encoding an enzymatically active carboxy-terminal portion of β-galactosidase. *Gene* 25, 71–82. doi:https://doi.org/10.1016/0378-1119(83)90169-5.

Svenningsen, S. L., Waters, C. M., and Bassler, B. L. (2008). A negative feedback loop involving small RNAs accelerates Vibrio cholerae’s transition out of quorum-sensing mode. *Genes Dev.* 22, 226–238. doi:10.1101/gad.1629908.

Urban, J. H., and Vogel, J. (2009). “A Green Fluorescent Protein (GFP)-Based Plasmid System to Study Post-Transcriptional Control of Gene Expression In Vivo,” in, 301–319. doi:10.1007/978-1-59745-558-9_22.

Will, S., Joshi, T., Hofacker, I. L., Stadler, P. F., and Backofen, R. (2012). LocARNA-P: Accurate boundary prediction and improved detection of structural RNAs. *RNA* 18, 900–914. doi:10.1261/rna.029041.111.

Yanisch-Perron, C., Vieira, J., and Messing, J. (1985). Improved M13 phage cloning vectors and host strains: nucleotide sequences of the M13mpl8 and pUC19 vectors. *Gene* 33, 103–119. doi:10.1016/0378-1119(85)90120-9.

Zhou, J., and Rudd, K. E. (2012). EcoGene 3.0. *Nucleic Acids Res.* 41, D613–D624. doi:10.1093/nar/gks1235.
